# An Experimental and Computational Study of the Effect of ActA Polarity on the Speed of *Listeria monocytogenes* Actin-based Motility

**DOI:** 10.1371/journal.pcbi.1000434

**Published:** 2009-07-10

**Authors:** Susanne M. Rafelski, Jonathan B. Alberts, Garrett M. Odell

**Affiliations:** Center for Cell Dynamics, University of Washington, Friday Harbor, Washington, United States of America; University of Notre Dame, United States of America

## Abstract

*Listeria monocytogenes* is a pathogenic bacterium that moves within infected cells and spreads directly between cells by harnessing the cell's dendritic actin machinery. This motility is dependent on expression of a single bacterial surface protein, ActA, a constitutively active Arp2,3 activator, and has been widely studied as a biochemical and biophysical model system for actin-based motility. Dendritic actin network dynamics are important for cell processes including eukaryotic cell motility, cytokinesis, and endocytosis. Here we experimentally altered the degree of ActA polarity on a population of bacteria and made use of an ActA-RFP fusion to determine the relationship between ActA distribution and speed of bacterial motion. We found a positive linear relationship for both ActA intensity and polarity with speed. We explored the underlying mechanisms of this dependence with two distinctly different quantitative models: a detailed agent-based model in which each actin filament and branched network is explicitly simulated, and a three-state continuum model that describes a simplified relationship between bacterial speed and barbed-end actin populations. *In silico* bacterial motility required a cooperative restraining mechanism to reconstitute our observed speed-polarity relationship, suggesting that kinetic friction between actin filaments and the bacterial surface, a restraining force previously neglected in motility models, is important in determining the effect of ActA polarity on bacterial motility. The continuum model was less restrictive, requiring only a filament number-dependent restraining mechanism to reproduce our experimental observations. However, seemingly rational assumptions in the continuum model, e.g. an average propulsive force per filament, were invalidated by further analysis with the agent-based model. We found that the average contribution to motility from side-interacting filaments was actually a function of the ActA distribution. This ActA-dependence would be difficult to intuit but emerges naturally from the nanoscale interactions in the agent-based representation.

## Author Summary

Cells tightly regulate the branched actin networks involved in motility, division, and other important cellular functions through localized activation of the Arp2,3 protein, which nucleates new actin filaments off the sides of existing ones. The pathogenic bacterium, *Listeria monocytogenes*, expresses its own Arp2,3 activator, ActA, in a polarized fashion and can thus nucleate dynamic actin networks at its surface to generate forces to move through the cytoplasm. This bacterium has thus served as a simplified system for experimental and modeling studies of actin-based motility. We use this bacterial system to quantify the relationship between ActA polarity and bacterial speed of motion by experimentally manipulating this polarity and analyzing the resultant ActA distributions and bacterial trajectories. Like many cellular behaviors, *L. monocytogenes* motility emerges from a complex set of biochemical and force-based interactions. We therefore probe this polarity-speed relationship with a detailed agent-based simulation which encodes the predominant biochemical reactions and whose agents (actin filaments, ActA proteins, and the bacterium) exchange forces. We contrast conclusions from this agent-based model with those from a simpler mathematical model. From these studies we assert the importance of a heretofore neglected force in this system – friction between actin filaments and the bacterial surface.

## Introduction

*Listeria monocytogenes* is a rod-shaped bacterial pathogen that can infect cells and spread from cell to cell directly, thus evading the host's normal immune response [1]. *L. monocytogenes* expresses the surface protein, ActA, which interacts with the host-cell actin-polymerization machinery, to propel itself through the cytoplasm in order to form membrane protrusions and move directly into a neighboring cell reviewed in [2],[3]. The ActA protein directly activates the Arp2,3 complex, which in turn nucleates branched actin networks at the surface of the bacterium [4]. ActA also interacts directly and indirectly with F- and G-actin, the cellular protein VASP, and profilin-actin reviewed in [2],[3]. The bacterium thereby harnesses the same dendritic actin array a motile cell deploys at its leading edge to create an actin ‘comet tail’ structure that propels the bacterium reviewed in [2],[3],[5].

The actin driven motility of *L. monocytogenes*, or of artificial cargo, is frequently used as a biophysical model system to understand the force-production mechanisms of actin-polymerization and the dendritic-actin array organization leading to cargo movement reviewed in [6]. Much of this experimental work has been done in an *in vitro* system in which *L. monocytogenes* move in cellular extracts or mixtures of purified protein components [7],[8]. Mathematical models of *L. monocytogenes* motility include those studying the contribution of bacterial, or filament, fluctuations on movement, and the actin-network as an elastic gel [9]–[11]. Recently, we created an agent-based simulation of *L. monocytogenes* motility, which recreated realistic bacterial motion by combining experimentally known rules and rates of biochemical interaction with a mechanism of force generation at the bacterial surface due to filament polymerization [12]. A modification of that simulation is our principal tool in this study. The resulting behavior of the *in silico* bacterium was an emergent property of the simulation and not one that could be directly predicted or controlled. The simulation, like the biological system, is ‘complex’ since global behaviors emerge in non-obvious ways from the encoded small-scale local interactions. Bacterial movement resulted from the combination of forward pushing forces due to actin polymerization and the tethering of filaments to the bacterial surface, ensuring the bacterium and the tail did not simply drift apart. Forward motion of the bacterium occurred due to the cooperative breakage of a set of tethers and led to a distribution of abrupt steps of nm sizes, which have recently been confirmed in experiments carefully tracking actin-propelled microspheres moving in extract [13]. In addition, the consideration of cooperative tether-breakage as the rate-limiting step for bacterial motility also has been subsequently experimentally supported [14]. This suggests that our complex simulation indeed replicated realistic mechanisms of force production in the *L. monocytogenes* system. Text S1, Fig. S1, and Tables S1 and S2 offer a more detailed explanation of the agent-based simulation, its assumptions, and its validation.

To understand the dominant force mechanisms regulating bacterial speed, we combine this simulation with new experimental results on ActA distribution patterns. A population of *L. monocytogenes* moving in the same extract system exhibits great variability in their steady-state speed [15]. Some of this variability can be explained by differences in the surface distribution of ActA protein, which nucleates new actin filaments, and thus can regulate the pattern of actin network growth at the bacterial surface. The ActA pattern on the surface of *L. monocytogenes* arises in a cell-cycle dependent manner [16],[17]. The typical bacterium has a higher ActA concentration on one pole than on the other, but as it grows and begins to divide, the opposite pole also accumulates ActA such that when the bacterium is ready to divide, both poles have high ActA density (bipolar ActA) relative to the center of the bacterium, which has the least [17],[18]. Thus, in each newly divided bacterium, ActA density is initially greatest at one pole, tapering off along the sides towards the other pole (unipolar ActA). Bacteria with more bipolar distributions were shown to move more slowly, due to competition between actin nucleation at both poles [17]. Within unipolar bacteria there exists a wide range of polarities with differences in the shape of the ActA distribution along the long axis of the bacterium [17]. In this study we address how the more subtle differences in ActA distribution in unipolar bacteria modulate bacterial speed.

It may seem obvious that concentrating more ActA at the ‘business end’ of the bacterium, where the polymerization it catalyzes most effectively moves it forward, would enhance bacterial speed. This turns out to be true, but for subtle reasons. Consider the following statements about the bacterial-actin interactions:

- The cylindrical geometry of the bacterium is important to its motion, with filaments nucleated at the sides contributing weakly, or not at all, to forward forces, while filaments nucleated at and/or interacting with the pole push the bacterium forward [17].
- Filaments generated at the sides grow autocatalytically, due to Arp2,3-dependent branching. Thus the sides provide many pre-polymerized filaments already integrated into the comet-tail actin network and primed to push when, due to forward displacement of the bacterium, they find themselves at the rear end.
- All filaments are also able to retard the bacterium due to their interactions with the ActA protein, either directly or possibly via Arp2,3 or Ena/VASP, which create transient forces tethering the tail to the bacterium reviewed in [3],[19].
- Filaments and the bacterium exchange kinetic friction forces proportional to the contact force of each interaction, though the importance of this force in determining motility is unknown.
- Motion of the bacterium feeds back onto this system –the slower the bacterium moves, the more filaments it can accumulate to generate propulsive forces, and vice versa.

The interplay between these competing propulsive and restraining mechanism ultimately determine the effect of ActA quantity and distribution on bacterial speed.

Here we experimentally alter the degree of polarity of ActA on the surface of *L. monocytogenes* and observe that the more polar bacteria move more quickly. To explore the mechanistic basis of this observation, we incorporate different ActA polarities into our agent-based model, which simulates all of the aforementioned competing forces. We find in our motility assay that ActA along the sides of bacteria principally slows bacterial speed, and that our simulation requires the incorporation of a cooperative restraining mechanism (i.e. a cooperative function of the number of filaments) to recapitulate this experimental observation. We suggest, due to the inherent cooperative nature of kinetic friction, that the friction forces between filaments and the bacterial surface, rather than the transient tether forces between filaments and ActA proteins or fluid coupling between filaments and the bacterium, are primary in determining how ActA polarity determines bacterial speed of motion.

## Results

### Bacteria with ultrapolar ActA distributions move faster

**Figure 1:**
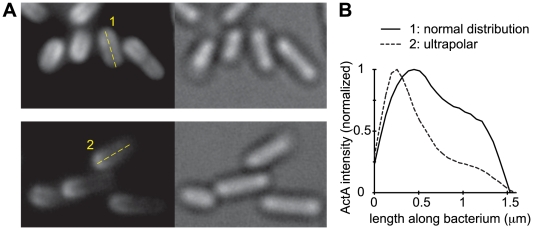
Ultrapolar bacteria display greater degree of polarity than normal bacteria. A) Images of normal (top) and ultrapolar (bottom) bacteria expressing ActA-RFP. Left panels show ActA signal, right panels show bright-field illumination. B) ActA intensity linescan of one normal and one ultrapolar bacterium labeled in A. The average intensity over the width of the bacterium is plotted at each point along its length. Intensities are normalized to the maximum in the linescan. Ultrapolar ActA bacteria display relatively less ActA along the sides than normal ActA bacteria.

We created populations of *L. monocytogenes* displaying a greater degree of ActA polarity than bacteria normally used in extract experiments (Fig. 1). Cell wall growth along the cylindrical body of the bacterium is faster than at the poles [18]. Thus, when bacteria with normal ActA distributions are rapidly grown for short periods of time, the protein is preferentially retained at the poles and more rapidly diluted along the sides, resulting in a greater degree of ActA polarity –we call these highly polarized bacteria ‘ultrapolar’ bacteria and contrast their motility with ‘normal’ bacteria. Linescans of ActA-RFP intensity along the length of the bacterium demonstrate that ultrapolar bacteria created in this manner display lower amounts of ActA along the sides with respect to the pole than bacteria with normal ActA distributions (Fig. 1) while the poles maintain comparable amounts of ActA.

Ultrapolar and normal populations were mixed to create a continuum of ActA surface distributions that we could directly compare within the same motility assay experiment. We performed time-lapse video microscopy of steady-state movement of these mixed polarity populations. To avoid confusion, we excluded bipolar ActA bacteria [17] in our subsequent analysis. Our final dataset included 253 individual bacteria from two separate experiments performed on the same day, using the same population of mixed polarity bacteria. In this paper, we confine our analysis to bacteria from a single population observed all on the same day to eliminate, as much as possible, variations in our ActA intensity and bacterial speed measurements that arise from experimental variation (e.g. differences in extract dilutions, temperature, etc). We saw the same trends, however, in additional independent experiments on several other days (data not shown).

**Figure 2:**
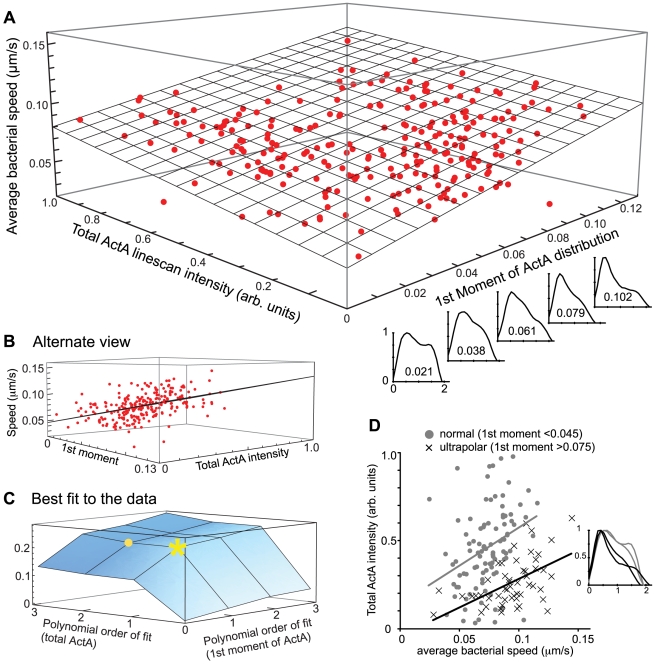
Bacterial speed increases with increased ActA polarity and intensity. A) 3-dimensional plot of the average experimentally measured bacterial speed vs. its 1^st^ moment and total ActA linescan intensity. The linescan graphs along the bottom right axis display the ActA distribution for respective 1^st^ moment values (intensity normalized to the maximum to highlight how the 1^st^ moment describes degree of polarity). The larger the 1^st^ moment, the greater the degree of polarity in ActA distribution. The total ActA intensities span a ten-fold range. Actual data points are shown in red. The best-fit plane is shown in gray. Speed increases with both ActA polarity (1^st^ moment) and ActA intensity. The bacteria that provided the prototypical normal and ultrapolar distributions used in simulations (Fig. 3A) are circled in black. B) A view of dataset shown in A from an alternate vantage highlighting the quality of the fit and scatter around the best-fit plane. C) 3-dimensional plot of the R^2^ residual values obtained when fitting the data shown in A by functions of increasing polynomial degree. The numbers on the x- and y-axes represent the highest polynomial degree of the function used for the fit. For example, the small round point at “2” on the ActA intensity axis and “1” on the 1^st^ moment axis represents the R^2^ value for the fit Z = a+bX+cX^2^+dY where Z is the speed, a, b, c, and d are fitting constants, X is the ActA intensity and Y is the 1^st^ moment (a similar plot representing the mixed non-linearities, i.e. where the yellow star would represent the function Z = a+bXY was also considered; data not shown). The greatest increase in R^2^ occurs for a linear fit in ActA intensity and polarity (yellow star) –higher degree polynomials only marginally improve the fit. This suggests that a linear fit in both 1^st^ moment and ActA intensity sufficiently describes the trend in the data. The plane shown in A and B corresponds to this fit. D) Speed vs. ActA intensity for ultrapolar and normal ActA bacteria. Bacteria were classified into ultrapolar and normal categories based on the value of their 1^st^ moment. Bacteria with 1^st^ moments below 0.045 and above 0.075 could be easily distinguished by eye. Bacteria with intermediate 1^st^ moments (between 0.045 and 0.075) were ignored in this analysis. Normal bacteria are shown as gray circles and ultrapolar bacteria as black crosses. Linescan inset contrasts two examples each of ultrapolar (black) and normal (gray) ActA distributions.

To obtain a continuous measure of the degree of polarity, we calculated, from measured ActA linescans, the 1^st^ moment of the intensities along the bacterium (normalized to bacterial length and maximum ActA intensity). The 1^st^ moment describes how asymmetric the ActA intensity linescan is around the center of the bacterium, with higher 1^st^ moments representing distributions with greater asymmetry (linescans on axis in Fig. 2A). The average speed of a bacterium was positively correlated to both the 1^st^ moment and to the total ActA linescan intensity (a measure of total ActA computed by integrating the ActA distribution over the surface of the bacterium) in this population (Fig. 2A and B). Therefore we sought a mathematical function giving the speed as a function of two independent variables (total ActA intensity and 1^st^ moment of ActA distribution). Approximations of this function as polynomials in the two variables will become arbitrarily accurate as the polynomial degrees increase. To ascertain how high the polynomial degrees should be before diminishing returns makes further increases in degree pointless, we generated fits to our measured data using all degrees less than 4. We found only slight increases in the R^2^ goodness of fit criteria above a fit linear in both 1^st^ moment and total ActA intensity (Fig. 2C). This suggests that the resulting best-fit plane (Fig. 2A) sufficiently describes the main trends in the data. The increases in speed as both ActA polarity and ActA intensity are increased individually and jointly are statistically significant (p = 7e-14, 1e-13, and 5e-8 both variables together and each separately, respectively). We randomized the data for each variable and performed multiple additional regression analyses to verify that no statistical trend could be found for the randomized data (p≫0.1). While our analysis demonstrates a clear dependence of speed on both ActA polarity and intensity, scatter about the best-fit plane in Fig. 2A suggests an underlying variability in average speed not explained solely by the ActA distribution.

We found we could easily distinguish, by eye, ActA distributions with 1^st^ moments below 0.045 from those with 1^st^ moments above 0.075, and thus categorized these bacteria into normal and ultrapolar classes respectively (linescans in Fig. 2D). We removed bacteria with intermediate 1^st^ moments (0.045–0.075) from this analysis in order to make a more stark comparison between bacteria with normal and ultrapolar ActA distributions. The average speed of bacteria was positively correlated to the total ActA intensity in both the normal and ultrapolar populations (p values 1e-3 and 2e-5 respectively; Fig. 2D). Further, the average speed of the entire ultrapolar ActA population (0.093 µm/s for 52 bacteria) was significantly greater than the normal ActA population (0.073 µm/s for 96 bacteria; p value 4e-8 by rank sum analysis; Fig. 2D), results that the polynomial fit in Fig. 2A represents. An analysis of the joint dependence of bacterial speed on polarity and ActA density (maximum linescan intensity), instead of total ActA, revealed the same statistically significant trends described above (data not shown). Whether two bacteria share the same maximum ActA density (implying that the less polar bacterium has greater total ActA) or the same total ActA (implying that the less polar bacterium has less ActA at its pole), the more polar bacterium moves at faster speeds, on average. Our results show that increases in the degree of ActA polarity increase the speed of *L. monocytogenes*, suggesting that the additional ActA along the sides in normal bacteria must have a slowing effect. Further, greater amounts of ActA lead to faster bacteria within the range of ActA intensities in these data.

### The agent-based simulation requires a cooperative restraining mechanism to replicate the observed polarity-speed dependence

**Figure 3:**
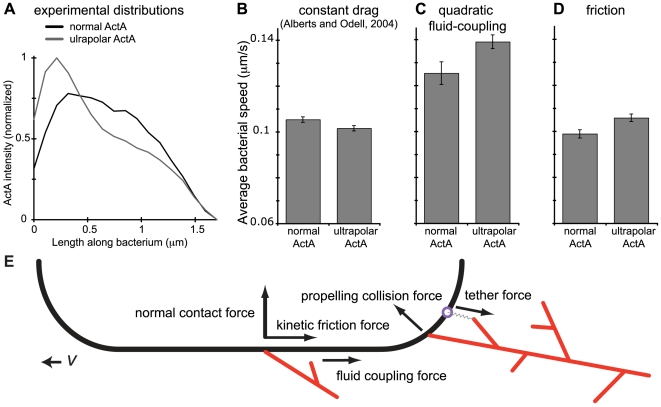
Ultrapolar bacteria move faster than normal ActA bacteria in simulations incorporating a cooperative restraining mechanism. A) Representative examples of a normal ActA distribution and an ultrapolar ActA distribution selected from the experimental dataset for use in the simulation. These distributions are scaled such that the total amount of ActA is equal. B) Representative set of simulation runs with normal and ultrapolar ActA distributions for simulations exhibiting a constant drag as in [12]. Regardless of the parameters chosen, the normal ActA bacteria always moved faster than the ultrapolar bacteria. In this particular set of runs, the average speed of the normal ActA and ultrapolar bacteria was 0.101 and 0.105 µm/s (n = 20 simulation runs for each distribution.) Average speeds were significantly different by rank sum analysis (p-value 0.038). C) Simulations runs with the identical parameters as in B but incorporating fluid coupling of the bacterium to the surrounding filaments with a quadratic dependence on filament number. Now the ultrapolar bacteria move more rapidly than the normal ActA bacteria (0.139 and 0.125 µm/s respectively, n = 15). Average speeds were significantly different by rank sum analysis (p-value 0.028). However, we cannot physically justify this quadratic fluid coupling (Fig. S2). D) Simulation runs with the identical parameters as in B but incorporating a frictional force between actin filaments and the bacterial surface. Ultrapolar bacteria move more rapidly than the normal ActA bacteria (0.106 and 0.099 µm/s respectively, n = 20). Average speeds were significantly different by rank sum analysis (p-value 0.005). This particular set of runs used a friction coefficient of 0.2. However these results were robust to changes in simulation parameters, e.g. for 6-fold higher friction coefficients and for simulations with increased actin tail densities (data not shown). Error bars are standard error. E) A schematic definition of the forces in the simulation shown on half the surface of a 2D capsule shaped bacterium. Any collision between a filament and the bacterial surface defines a contact force, assumed normal to the bacterial surface regardless of filament orientation. The collision may be the result of thermal motions of the bacterium and actin structures, or a filament may polymerize ‘into’ the bacterium. Bonds between actin filaments and surface bound ActA proteins can lead to a tether force if that bond is strained. This protein-protein tether is distinguished from the smaller scale forces between electrically charged molecules that lead to kinetic friction. A shape-based viscous drag is assumed for all bodies in the simulation, but that drag might be modified to account for the fluid-coupling between bodies. To include this fluid-coupling between actin filaments and the bacterium we increase the bacterial drag as a linear function of the surrounding filament population.

To explore the mechanisms by which polarity affects bacterial speed, we incorporated into the simulation examples of both normal and ultrapolar ActA distributions from our experimental dataset with comparable total ActA intensities (Fig. 3A). In both the original version of the simulation [12] and in our updated version (see Materials and Methods) ultrapolar ActA bacteria consistently moved more slowly than normal ActA bacteria (Fig. 3B, “constant drag”). The faster speed our initial model predicted for normal ActA bacteria is due to the greater density of actin filaments generated along the sides of these bacteria compared to the lower density of these filaments along the sides of ultrapolar bacteria. The role of side-generated filaments in enhancing speed is can be demonstrated with simple, artificial ActA distributions. Simulations in which the ActA is fully confined to the poles produce much slower bacterial movement than if a small amount of ActA (20% of total) is distributed on the sides (data not shown). These side-generated filaments enhance speed by creating larger branched actin networks that will produce greater forward forces, once they come into contact with the rear bacterial pole. These side-generated filaments may also interact with many different ActA proteins as the bacterium moves past them, and they are thus protected from capping or actively uncapped. For this reason, filaments interacting with the bacterial sides are, on average, the longest-lived filaments. The simulations produce a large distribution of filament lifetimes with very many short-lived filaments –largely those that diffuse away from the bacterium and are quickly capped. But more than half of the filaments persist for longer than 10 seconds and almost a third persist for longer than 20 seconds (data not shown). For a 1.7 µm long bacterium moving at ∼100 nm/s, this is plenty of time for a side-generated filament to enter the population of filaments interacting with the bacterial pole.

On the other hand these same filaments form tethers with the bacterial surface, via ActA, and thus also generate pulling forces retarding bacterial motion. Since the population of filaments along the sides of the bacterium can only restrain the bacterium in our model (pushing by filament tips is assumed to be in a direction normal to the bacterial surface), we initially reasoned that ultrapolar bacteria might be made to move faster than normal ActA bacteria if we simply changed the nature of the actin filament-bacterial surface tethers, allowing side-filaments to retard motion more than they enhance motion in a filament number-dependent fashion and thus slowing normal ActA bacteria more than ultrapolars. Extensive parameter searches, varying tether toughness, breakage criteria, and number (by adjusting the parameters governing new and autocatalytic branch creation) failed to find parameter sets matching our *in vivo* observations. Increasing tether number or strength per tether (1000-fold range) did slow the overall speed of both normal and ultrapolar ActA bacteria. However, the normal ActA bacteria were always faster than the ultrapolar bacteria up until the point that the tether number or strength was great enough to stall the bacterial motion altogether (data not shown).

We additionally explored values of other simulation parameters that might affect the polarity-speed dependence, including parameters affecting actin growth (actin nucleation (50-fold range), depolymerization (4-fold range) and branching rate (25-fold range)), strength of the attachments between the comet tail and its surroundings (50-fold range), and the viscosity of the environment (6-fold range). These parameters did affect the overall speed of bacteria, and the nature of the trajectories (e.g. smooth vs. hoppy motion), and some produced simulation runs in which ultrapolar ActA bacteria moved almost as fast as normal bacteria. However, ultrapolar ActA bacteria consistently moved more slowly than normal ActA bacteria (Fig. 3B, “constant drag”) and, despite extensive searching, we found no parameter set that produced the speed-polarity relationship we observed experimentally. This suggested that the simulation required filament-dependent restraining forces of a different nature.

We introduced a representation of both fluid coupling between the bacterium and the actin network around it and a representation of friction forces between individual filaments and the bacterium (Fig. 3). These forces add realism to the model; their existence is unquestionable. Only when we used a cooperative restraining mechanism, i.e. a restraining force that increases more than linearly with the number of filaments, could we replicate the experimental ActA polarity-speed dependence, obtaining simulation runs in which ultrapolar ActA bacteria move faster than normal ActA bacteria (Fig. 3C and D). With fluid coupling of the bacterium to the surrounding filaments, we can create, by making up a formula, such a cooperative restraining force (Fig. 3C), but we cannot justify it physically (Fig. S2). Thus realistic fluid coupling (i.e. non-cooperative coupling) does not reproduce our experimental results. Kinetic friction between the bacterial surface and side filaments is, on the other hand, inherently cooperative. The kinetic friction force between a filament and the bacterium is proportional to the contact force between them, i.e. 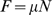 where 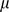 is the coefficient of friction and 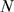 is the normal force. But additional side filaments polymerizing at the bacterial surface cooperate to increase the average normal force 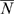, i.e. 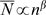 (Fig. S2) where 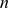 is the number of contributing filaments and 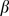 is an unknown factor of cooperativity. The total frictional drag force is just a summation of the contributions from each of the 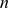 filaments and is approximately 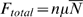, or 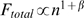, i.e. frictional drag restrains that bacterium cooperatively as a function of filament number by the factor 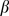. Such a frictional force was incorporated into the simulation by specifying a non-zero friction coefficient, 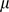, for filament-bacterial surface interactions. For sufficiently large coefficients of friction (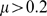), this robustly led to greater speeds for the ultrapolar bacteria than the normal bacterial (Fig. 3D and see Video S1 for representative examples of a normal and an ultrapolar ActA bacterium). Note that in the agent-based simulation the value for 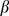, which depends on particulars of the filament population (e.g. filament and branch drags forces and orientations), emerges from the many individual filament-bacteria interactions. We find average values of 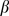 between 0.6 and 0.7 (Fig. S3B).

**Figure 4:**
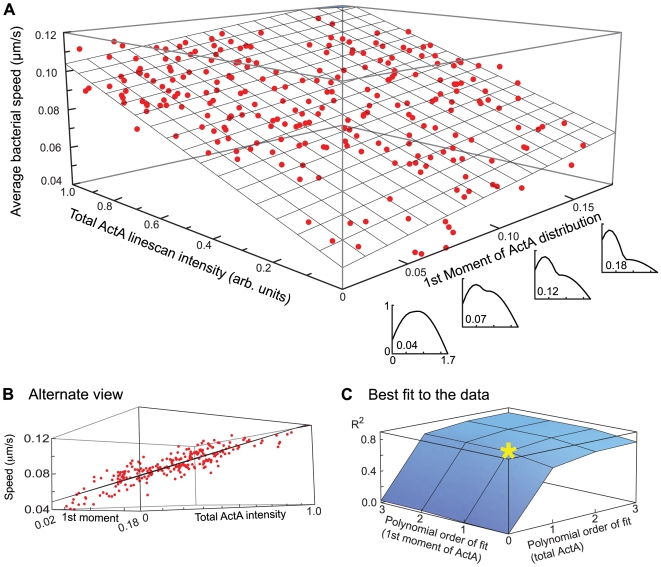
Bacterial speed increases with increased ActA polarity and intensity in simulations that incorporate friction. A) 3-dimensional plot of the average bacterial speed vs. its 1^st^ moment and total ActA linescan intensity for 253 simulated bacteria. A range of simulated ActA polarities was chosen to correspond to the experimentally observed range (Fig. 2A). Similarly, the total ActA intensities were set to span a ten-fold range as in the experimental data. The linescan graphs along the bottom right axis display the ActA distribution for respective 1^st^ moment values (intensity normalized to the maximum). Actual data points are shown in red. The best-fit plane is shown in gray. B) An alternate view of the dataset shown in A, highlighting the quality of the fit and scatter around the best-fit plane. C) 3-dimensional plot of the R^2^ value obtained when fitting the data shown in A by functions of increasing polynomial degree. As in Fig. 2C the greatest increase in R^2^ occurs for a linear fit in ActA intensity and polarity (yellow star) –higher degree polynomials only marginally improve the fit.

With this qualitatively realistic frictional drag force by side filaments, our simulation replicated the polarity-speed dependence of two specific experimental ActA distributions. However, our experimental results indicate a dependence of speed on both ActA polarity and on total ActA intensity. To test this experimental result further in our simulation, we constructed an ad-hoc mathematical function, as the sum of two sine waves with one varying parameter (Fig. S4), to create artificial ActA distributions that span the range of our experimental measurements. In this way we could easily generate a large simulation dataset as the *in silico* analogy to the experimental data (compare Fig. 2 and Fig. 4). In this simulated dataset, the average speed of a bacterium was positively correlated to both the 1^st^ moment and the total ActA intensity (p = 8e-80, 4e-19, and 2e-80 both variables together and each separately, respectively). This suggests that our simulation, by incorporating a frictional force between actin filaments and the bacterium, captures the experimentally observed speed dependence for a continuum of polarities and intensities.

### A simple mathematical model describing the simulation results

The nanoscale details that lead to microscale *L. monocytogenes* motility are complex. Individual filaments are created as branches from filaments oriented at any angle to the bacterial surface. These actin branches form cross-links to each other and to other filaments and bodies in the cell environment, thereby gaining purchase from which polymerizing actin barbed-ends can push the bacterium forward. The behavior of any individual bacterium is influenced by the stochasticity of the various biochemical events, by the Brownian motion to which all cellular bodies are subject, and by the particular location of individual ActA proteins on the bacterial surface. *In silico*, we have tried to capture these nanoscale details and mechanisms, and have succeeded in building a simulation whose emergent motility is much like that of the actual bacteria [12].

**Figure 5:**
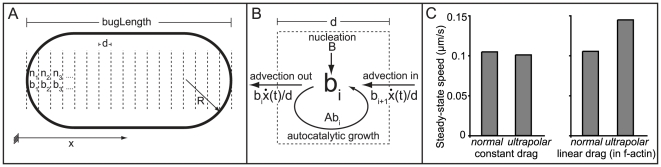
A one-dimensional barbed-end tracking model of *L. monocytogenes* motility. A) The bacterium is spatially discretized into 0.1 µm long mesh elements along the long-axis (the x-direction). In each of the mesh elements, two state variables 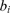 and 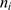 –the number of barbed-ends and the f-actin density, respectively– determine the element contribution to propulsion force and drag coefficient. B) Equations for 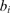, for example, are derived from conservation of barbed ends at a single mesh element: 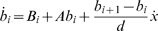, where 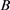 is the nucleation of new barbed-ends (i.e. a new mother filament), 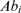 is the autocatalytic creation of barbed-ends from existing barbed-ends (i.e. branching), and 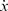 is the velocity of the bacterium. Only elements on the hemispherical cap significantly contribute to propulsion force, while f-actin along the side of the bacterium contributes to the drag force. The ratio of force to drag determines the instantaneous velocity 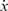. A similar element diagram can be drawn for 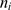, though without any autocatalytic term. C) Steady state speeds for different ActA distributions. A constant drag on the bacterium led to faster normals than ultrapolars. Incorporation of a linear filament-dependent restraining mechanism, representing either (or both) filament-surface tethers or fluid coupling led to faster ultrapolars than normals, as in our experimental observations. A cooperative filament-dependent restraining mechanism (representing kinetic friction, for example) similarly led to faster ultrapolars (data not shown).

But it is a fair criticism to point out that the simulation, due to its very complexity, doesn't build intuition. We have thus encapsulated the major mechanisms from our complex model into a vastly simpler one-dimensional continuum model (Fig. 5, Text S2), formulated as a set of partial-differential equations (PDEs) with state variables of actin barbed-end number, actin density, and speed of motion. We built this continuum model to compare and contrast its predictions and robustness of behavior with the agent-based simulation.

In the solution of this model the bacterium is spatially discretized into a one-dimensional set of elements, each of size 0.1 µm, spanning the bacterium at the optical resolution of our experimental images of ActA distribution and bacterial motility (Fig. 5A). Thus our measured ActA distributions can be directly mapped onto these elements. The model tracks through time the barbed-end and f-actin populations in each mesh element (Fig. 5B). Bacterial velocity and drag coefficient are constructed as simple functions of this barbed-end population. New barbed-ends are created (de-novo in the simulation, although this represents both de novo nucleation and the capture by ActA of small f-actin fragments) in each spatial element as a function of the number of ActA proteins in that element, and also autocatalytically, in proportion to the number of barbed-ends. Filamentous actin growth is also proportional to the number of barbed-ends in each element. Barbed-ends and f-actin flow into, and out of, each mesh element at a rate dependent upon the speed of the bacterium. We assume that, as in the complex model, only barbed-ends in contact with the hemispherical caps can effectively push the bacterium forward, so the propulsion force, 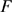, is a function of barbed-ends in the elements near the back end of the bacterium. The drag coefficient, 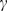, is dependent on the f-actin populations summed up along the length of the bacterium. The instantaneous velocity of the bacterium is 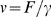. As this model is formulated in terms of average quantities (e.g. average barbed-end creation rates, average force per filament) we can justify some of the parameter values through analysis of the equivalent emergent property in the agent-based model (Text S2).

The predictions of this model are coarsely consistent with our results from the agent-based model, but less specific about the nature of the restraining forces. The differential-equation model predicts that ultrapolars will move faster than normals with any restraining mechanism that is filament number dependent, whether linear or cooperative (Fig. 5C). This means that this simple model can explain our experimental observations as an effect of either the protein-protein ‘tether’ bond between ActA and actin filaments, fluid coupling between the bacterium and actin filaments, or friction. Therefore, our continuum model has limited resolution as an investigative tool for this study. To be clear, this simple model is not inconsistent with the posited role for kinetic friction, it just does not require such a cooperative restraining mechanism to replicate the experimental results. We explore the reasons for the discrepancy between models in the Discussion.

## Discussion

Our experimental observation of how the speed of *L. monocytogenes* depends upon ActA distribution sets an important constraint for any model of that system: bacterial speed should increase, up to a point, with both ActA amount and the polarity of the ActA distribution. Mathematical modeling can determine the importance of forces acting on bacteria by exploring the underlying mechanisms that satisfy this constraint. We have attempted this with two models, a complex agent-based simulation and a far simpler partial-differential equation representation. We find that the partial-differential model can capture the coarse behavior of the biology, but that it is insensitive to the details of the restraining forces we wish to explore. Specifically, the partial-differential equation model produces the correct ActA distribution-speed relationship if the bacterial drag is assumed dependent upon actin filament number. Further, the mathematical form of this drag is unimportant in this model –the model can be made to satisfy that constraint through quantitative parameter tuning for many qualitatively different assumptions about the functional shape of this drag (i.e. linear vs. cooperative). Thus, our continuum model cannot distinguish between, or reach conclusions as to the relative importance of, filament-ActA tethers, fluid coupling, and kinetic friction between filaments and the bacterial surface. Our continuum model is not entirely wrong, but it is the wrong model for the questions we have posed. The agent-based simulation, however, leads us to a qualitative conclusion about the biology by distinguishing between these mechanisms. We conclude that a cooperative restraining force operates in determining motility characteristics and that this force likely arises from the kinetic friction between filaments and the bacterial surface.

We explored force-based mechanisms that lead to ultrapolars moving faster than normals, but there are reasonable biochemical explanations as well. The most obvious of these is actin monomer depletion at the rear of the bacterium. If the denser actin growth from normal ActA distributions leads to substantially greater actin monomer depletion, relative to the ultrapolar bacteria, then slower speeds for normal bacteria could result simply from decreased polymerization. Estimates (Text S1, [20]) suggest that the monomer flux to the bacterial surface, using the measured diffusivity of actin monomer through an actin gel [21],[22], is sufficient to replenish monomers used in polymerization. There is not unanimous agreement on this point, however [23]. Another biochemical possibility is that the local actin-filament nucleating power of ActA may depend cooperatively, rather than just linearly, on local ActA concentration. Distributing the same number of ActA molecules in an ultrapolar vs. normal pattern would then change the resulting total rates and locations of actin filament initiation and that alone could make ultrapolars move faster. We have not rejected this possibility, and indeed, there is new experimental evidence implicating it [24]. Such a hypothesis can be tested in our agent-based model: each ActA protein in the simulation can determine its nearest neighbors and then have enhanced Arp2/3 activation if those neighbors are sufficiently close. Footer et al. [24] propose two distinct pathways to explain the observed ActA cooperativity –we propose as a future direction to test these two possibilities *in silico*.

Additionally, neither the agent-based or continuum models we present explicitly consider gel effects, and they thus ignore the mechanisms proposed in elastic propulsion models [10],[25]. In the elastic propulsion model for bacterial motility, strain energy induced in the actin network surrounding the bacterium by continued actin growth at the surface is released by “squeezing” the bacterium forward and out of the actin shell. Increased computational power since this work began will allow our next generation model to simulate flexible, crosslinked, and entangled filaments; an approximation of gel effects will emerge naturally in this model. But our present model cannot address the importance or impact of this mechanism to bacterial motility. It is possible that gel effects might lead our model to different qualitative conclusions concerning the importance of the considered restraining mechanisms, i.e. a lesser role for friction. We suspect, however, that our current model, in ignoring the large hoop stresses that develop in the actin gel about the bacterium, underestimates the filament-bacterium contact forces. Higher contact forces would suggest an important role for friction with friction coefficients lower than we have used here.

Our experimental dataset spans a large range of different ActA amounts and polarities (1^st^ moments). We explored *in silico* behaviors with ‘normal’ and ‘ultrapolar’ ActA bacteria (representative distributions near the extremes of our measured ranges) and with mathematically generated distributions to span this entire range. Normal and ultrapolar ActA distributions (Fig. 3A) create different actin populations about a bacterium at steady-state motion. Any filament created on the side of the bacterium will immediately contribute to an increased drag on the bacterium through both fluid coupling and frictional drag when in contact with the bacterial surface. This filament may additionally participate in the creation of new daughter branches through its interactions with ActA on the bacterial surface, accompanied by a restraining tether force between the filament and the bacterial surface. This filament (and its daughter barbed-ends) will eventually advect, relative to the bacterium, to become part of the population of barbed-ends that can propel the bacterium forward by pushing at the rear. Therefore, relative to ultrapolars, normal ActA distributions create more robust actin populations along the sides that can simultaneously feed the propulsion machine with polymerizing barbed-ends, and restrain the bacterium through tethers, increased effective drag, and kinetic friction. Whether the net result of these competing effects is an increase or decrease in speed, relative to some standard distribution, depends on the qualitative details of the distribution (i.e. the 1^st^ and potentially higher moments). From our results with the agent-based model, we explain the ActA polarity-speed dependence of *L. monocytogenes* as the result of these several competing and interacting dynamic subsystems in which a cooperative restraining mechanism, likely the frictional force between actin filaments and the bacterial surface, determines how ActA polarity affects bacterial speed. The cooperative nature of this restraining mechanism shifts the balance between pushing and pulling such that filaments along the cylindrical sides of the bacterium hinder forward motion more than benefit it.

*In vivo*, *L. monocytogenes* is a large object moving through a cytoplasm densely packed with cytoskeletal structures and other cellular bodies. It is well known that there is a non-linear size-effect on the diffusion of cytoplasmic bodies [26],[27]. While very small proteins may diffuse as if in water, a large object like a bacterium would have an effective diffusion coefficient many times less than their shape-based values in water because any movement, causes, and is impeded by, collisions with myriad cellular inclusions. Our earlier model dealt with this cellular reality with a constant scaling-down of the shape-based diffusion coefficients (typically by 1/3000). In consideration of the fluid coupling that must exist between the bacterium and the filaments that form around it, we alternately explored the effect of varying this scaling constant as a function of the actin population along the bacterium. Such a scheme captures the (previously absent) realism that *L. monocytogenes* affects it's own viscous environment, but fails to satisfy our experimental constraint that ultrapolars outrun normals.

Of the restraining forces shown in Fig. 3E, only friction is cooperative. As detailed in Fig. S2, we expect the restraining force from both the ActA-filament tether force and any fluid coupling mechanisms to increase linearly, at most, with the number of actin filaments about the bacterium. For example, no matter the tether parameters, 100 tethers should exert (on average) a restraint force that is 100 times that of a single filament. Likewise, fluid coupling under Stokes flow can justify only a linear increase (again, on average since we are neglecting filament orientation in this argument) in drag on the bacterium with increasing actin filament number. At higher filament densities we would even expect this drag to increase more slowly, e.g. two nearly coincident filaments will not double the drag of a single filament. Unlike restraint by ActA-filament tethers or fluid coupling, restraint by friction forces is inherently a cooperative process (see Results and Fig. S2).

The only filament-number-dependent restraining force considered in other *L. monocytogenes* motility models has been an assumed net result of the many transient ActA-filament tethers [10],[11]. This tether force and the bacterial drag force are typically the only forces that oppose bacterial motion considered in past models; to our knowledge, this is the first suggestion of a role for kinetic friction between filaments and the bacterial surface. Though friction is familiar enough to us in the macroscopic world, it is reasonable to question the nature of this force for nanoscale contacts between biological materials. Specifically, we have assumed that Amontons' law applies for contact between filaments and the bacterial surface, i.e. that the magnitude of the friction force is proportional, through a friction coefficient, to the contact force normal to the direction of motion. The nature of friction at the microscopic scale is not fully understood, but research in this field largely supports our assumption [28]–[30].

The slower, normal ActA distribution is, indeed, the “normal” (i.e. typical) distribution for *L. monocytogenes*. That a *L. monocytogenes* doesn't usually move as rapidly as it might with a more polar ActA distribution is likely indicative of the task before it in infecting neighboring cells: the bacterium is more of a bulldozer than a racehorse, which has been observed powering through cellular bodies such as mitochondria with little change in speed [31]. The additional resistance from mitochondria in the path could be small, compared to the restraining forces on a bacterium from the dense shell of actin filaments it has catalyzed.

### Suitability of modeling methodologies

The qualitative differences between a simple continuum model and the complex agent-based model motivate a discussion of biological modeling methodologies in general. These two models were constructed under different design principles – the partial-differential model was constructed with a single narrow question in mind, while the agent-based model, designed to incorporate a large degree of low-level “realism”, can address many different questions. The failure of our particular continuum model to exhibit qualitative differences in outcome corresponding to qualitative differences in restraining mechanism does not denigrate analytical models in general –a modification of Gerbal et al. [10] in which friction force is made proportional to the radial actin-gel pressure might reach similar conclusions as our agent-based model, for instance. The point here, however, is that our initial best-intuition continuum (mean field) model doesn't have sufficient resolution to uncover the reason ultrapolar ActA distributions lead to faster bacteria. Absent insight from the agent-based model we would have overlooked the necessity of a cooperative restraining force, in the form of cooperative kinetic friction.

Why do the agent-based and continuum models reach different conclusions? We assert that the average relationships of the continuum model mask a smaller time-scale process critical to the correct emergent behavior. Thus, we find that the parameters chosen as constants in the continuum model are, in reality, functions of ActA distribution, our experimental variable.

To construct our continuum model, which tracks the dynamics of three state variables (barbed-ends, filamentous actin, bacterial speed) through time and in one-dimensional space, we have to assume some average relationships. We assume: an average propulsive force for each filament on the rear hemispherical cap of the bacterium, an average restraining force per unit actin (for fluid coupling) or per barbed-end (constant for ActA-filament tethers or filament number dependent for kinetic friction), an average autocatalytic and de novo barbed-end creation rate, and an average f-actin growth rate. We found that the continuum model is insensitive to the nature of the three restraining mechanisms we have modeled: fluid coupling between filaments and the bacterium, ActA-filament tethers, and kinetic friction. We interpret this insensitivity to mean that behaviors critical to the correct emergent ActA distribution response occur on a time-scale for which the assumed average relationships of the continuum model are not valid. Consider a filament born 1 µm from the rear tip of the 1.7 µm long bacterium, a bit forward of the centroid. If the bacterium moves at 100 nm/s (a typical speed) then it might interact with the bacterium for as long as 10 seconds before becoming part of the trailing actin tail. During that interval this filament may undergo many polymerization, collision, ActA tethering, and branching events. In other words, filaments stochastically sample a large number of possible states during their lifetime, and this is especially so for the filaments that interact the most with the bacterium. For instance, the ability of a filament (or branch structure, if it is part of one) to impart large forces on the bacterium generally increases until the bacterium has long passed (see actin distribution Fig. S5). It is this process –the stochastic maturation of a filament– that the average parameter assumptions of our continuum model have no way to represent. We support this claim by using the agent-based model to determine the time-scales at which our continuum model assumptions are reasonable. Average relationships begin to emerge when we average the agent-based data over several seconds, but are only clearly valid on time-scales of 10 seconds or more –a hundred thousand time-steps in the agent-based model (Fig. S3). We can also use the agent-based model to demonstrate that the maturation states, and thus appropriate average contributions to bacterial propulsion and restraint, of any side-interacting filament depend on the ActA distribution. Therefore, relationships assumed constant in the continuum model are actually functions of the experimental variable. We explicitly show this for two parameters in the continuum model: the propulsive force per filament and the autocatalytic barbed-end creation rate (Fig. S3B).

Based on our analysis with the agent-based model, we postulate that it might be possible to rectify the discord between models by introducing a third independent variable, filament age, into the PDE description. This modified continuum model would then track barbed-ends and filamentous actin in time, 1D-space, and age, thus allowing a filament age-dependent formulation of propelling and restraining forces, and perhaps of branching and elongation rates. We could use the agent-based model to establish the average age-dependence (as in Fig. S3B) of these parameters, but the revisionist tinkering required to compensate for the time-scale insensitivity of the continuum model undermines its usefulness, for this study at least. We prefer the model in which these dependences simply emerge, and our long-term interests lie with the building of realistic nano-scale encoded simulations that can address a large number of biological questions. But while we do not pursue a fix for this particular continuum model in this context, it should be remarked that simplified microscale models of inherently nanoscale processes are absolutely necessary to address certain biological questions. Consider a modification of our ActA distribution study focused on actin network behavior at the leading edge of a large motile cell. In that case we might attempt to characterize actin growth as a function of the density and distribution of nucleation promoting factors (i.e. Arp2/3 activators) on the cortex. The computational cost of using an agent-based model similar to ours to track every actin filament at the periphery of this cell is, at present and in the foreseeable future, prohibitive. A microscale model that provided an appropriate synopsis of the actin network behavior might, however, succeed on the whole cell scale. As demonstrated in Fig. S3, a nanoscale agent-based model can establish parameter values for application in a microscale model.

As a last word, we believe that the historical narrative of our work can serve as a parable for others. We described extensive parameter searches with the agent-based model, when it lacked a cooperative restraining mechanism, that all failed to match our experimentally observed polarity-speed relation. This iterative process was very time-consuming, as simulation of a single bacterium (representing one parameter set) requires several days of computer time. Ultimately, we found that this model produced robustly wrong behaviors, and this pointed to its lacking a critical mechanism. With the inclusion of kinetic friction, the model robustly predicts that ultrapolar bacteria move faster than normals. We might have saved ourselves many months searching through parameter space to find values that might make a qualitatively wrong model yield predictions that agreed with our *in vivo* data had we heeded a principle we now believe should guide biological modeling: Biological systems have evolved to do what they do robustly, so emergent behaviors arise from the system topology/connectivity and should not depend on a precise and fragile balance of the parameters characterizing interactions between molecular parts or in the relative abundance of those parts (i.e. the concept of structural stability in mathematics). Had we succeeded in finding a parameter set for the agent-based model that matched our experimental constraint, that result would have been suspect due to the very difficulty in finding it.

## Materials and Methods

### Bacterial growth conditions to generate ultrapolar ActA distributions

*L. monocytogenes* displaying a greater degree of ActA polarity (ultrapolar) were created by manipulating bacterial growth conditions. Bacteria expressing normal, wild-type ActA distributions (achieved either by use of a constitutively expressing strain or by ActA induction) can be made more polar by growing them rapidly for short periods of time. While ActA has a long residence time on the surface of bacteria on the order of several hours [18], some of the ActA protein is still lost from the entire surface during the growth to create the ultrapolar distribution. Thus each combination of initial ActA expression level and rapid growth conditions will result in a different range of ActA distributions and intensities within the population. For these experiments we used the following empirically determined conditions for the optimal combination of polarity and minimal ActA loss. *L. monocytogenes* strain JAT-395 [17] expressing ActA-RFP under the wild-type ActA promoter was induced to early stage IV in ActA polarization [18] (ActA distribution almost the same as in constitutively expressing strains). Bacteria were then diluted 10-fold into BHI and grown one hour (approximately one doubling time), then used in motility assays.

### Motility assays and microscopy

JAT-396 bacteria (constitutively expressing ActA-RFP) [17] were grown for 9 hours with shaking at 37°C in 5 mL LB containing 7.5 µg/mL chloramphenicol. These bacteria display the previously described normal polar ActA distributions [17]. To analyze both ultrapolar and normal bacteria simultaneously, bacteria from both populations were mixed at a 2∶1 ultrapolar∶normal ratio. The mixture was spun down and re-suspended in Xenopus buffer (XB) [32] to an O.D._600_ of approximately 9.0 then used continuously in multiple independent motility assays for 5 hours (maintained on ice; *L. monocytogenes* in XB remain alive but no longer grow and thus maintain their ActA distribution during this time).

*L. monocytogenes* in vitro motility assays were performed as described [17]. 25 µL *Xenopus laevis* egg extract, 2.5 µL ATP regenerating mix [32], and 2 µl of rabbit muscle AlexaFluor488 labeled actin (diluted to 1.1 mg/mL, 1.5 dyes/actin; Invitrogen, Carlsbad, CA) were mixed and diluted with XB such that the final motility assay was 50% of the original extract concentration, then kept on ice. 1 µL resuspended bacteria and 1 µL 0.9 µm prediluted silica spacer beads were added to 5 µl extract mixture. 1.2 µL of the mixture were immediately spread between a glass slide (Gold Seal, Portsmouth, NH) and 22 mm, #1 square coverslip (Premium Cover Glass; Fisher Scientific, Hampton, NA), sealed with VALAP (vaseline∶lanolin∶paraffin; 1∶1∶1) and used for imaging of steady-state motility.

Microscopy was performed on an Olympus IX70 equipped with an x-y-z automated stage (Applied Precision, Issaquah, WA) and a cooled CCD camera (CoolSNAP HQ; Photometrics, Tucson, AZ). Timelapse images were taken using a 60×, 1.4NA PlanApo lens and collected every 5 s for 2 minutes using Softworx software (Applied Precision, Issaquah, WA).

### Bacterial tracking and parameter measurements

Bacteria were tracked at their centroid using the semi-automated threshold dependent “track objects” function in Metamorph (Universal Imaging, Downington, PA). Tracked bacteria were imaged between 1.5 and 2.5 hours after mixing with extract to ensure this analysis included only bacteria moving at steady-state. Bacteria displaying bipolar ActA distributions [17] were not included in this dataset. Bacterial tracking was performed separately from ActA linescan measurements and the data eventually recombined. The ActA linescan intensities were measured in ImageJ with the plot profile function as follows. The background intensity for a fluorescent image of ActA was determined as the average mean intensity of several large rectangular measurements (Rectangular Selection Tool: Analyze→Measure); we subtracted this intensity from the image. ImageJ's Straight Line Selection tool was used to determine the length of the bacteria on the bright-field image. A straight line of this length, with an averaging width of five pixels, was centered as well as possible (given the coarse pixilation) on the fluorescent image to obtain a plot profile of ActA intensity along the length of the bacterium (Straight Line Selection Tool: Analyze→Plot Profile).

### Analysis of ActA polarity

To generate a continuous measure of ActA polarity (from least to most polarized) the ActA linescan was used to calculate the 1^st^, 2^nd^, etc. moments by calculating:
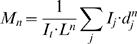
where *M_n_* refers to the *n*th moment, *L* = length of bacterium, *I_t_* = total linescan intensity, *I_j_* = linescan intensity at pixel *j* (i.e. j-th discretized mesh point) along bacterium, and *d_j_* = distance from pixel *j* to center of bacterium. A principle component analysis of the data was performed (using JMP; SAS Institute, Cary, NC) which showed that the 1^st^ moment was the most significant of the first 5 moments (0^th^ to 4^th^ moment) for describing the experimental polarity-speed dependence. The 2^nd^ moment is useful to differentiate between unipolar and bipolar ActA bacteria. However, since all bacteria with bipolar distributions were removed from the dataset, this measure was not critical to our analysis. The total amount of ActA on a bacterium was determined as the sum of the intensities at each camera pixel in the linescan (also the same as the 0^th^ moment).

No correlation between speed and bacterial length was found (data not shown), but to ensure no effect from extremely long or short bacteria, the final analyzed datasets were limited to bacteria between 10 and 25 pixels (1.06 and 2.26 µm) in length (>90% of tracked population). The final filtered dataset shown in Fig. 2A included 253 bacteria. Statistical significance for the difference between the average speeds for this analysis was determined using rank sum analysis. The Statistical significance of the linear correlation was calculated with the “regression” analysis tool in Microsoft Excel.

3-dimensional plots showing average speed per bacterium as a function of 1^st^ moment (polarity) and total ActA were created in Mathematica (Wolfram Research, Champaign, IL). To determine which polynomial functions best fit the data, the R^2^ values from different polynomial-order fits were plotted; the improvement in fit beyond linear was minimal. The statistical significance of the linear fit was calculated with the “regression” analysis tool in Microsoft Excel. The values within each category (i.e. average speed etc.) were randomized and recombined. A regression analysis of these randomized data confirmed no significance in the linear fits (p≫0.1).

### Computational Methods

We made two different computer models. One is a nanoscale-detail-oriented stochastic model of the biochemical and force-based interactions between a rigid *in silico L. monocytogenes* and the actin filaments/branches whose creation that bacterium catalyzes. A description of this agent-based model can be found in Alberts and Odell [12], with some distinctions described here. The Java source code for this model, RocketBugs, is available at www.celldynamics.org→downloads→simulation code. The second model is a simple partial-differential equation formulation of a mean field theory, informed by our explorations with the more complex model; a derivation of this PDE model can be found in Text S2, and its numerical solution is implemented in Text S3, a Mathematica notebook.

The stochastic model is computationally intensive, with typical run-times (for 6 minutes of simulated time) of three to five days, depending on parameters, on modern Linux servers running Java 1.6. These simulations additionally require up to 2 GB of main memory per server, again depending on the particular parameter set.

### Changes to the stochastic model from Alberts and Odell [12]

#### Filament drag

The method by which we simulate the interactions of filaments/branches with each other, and with other cellular entities not explicitly simulated, has changed greatly. In Alberts and Odell [12], the assumed viscous drag of an actin filament/branch increased greatly as a simple function of its age; after a threshold drag was attained, the filament/branch was simply assumed to be fixed in space. This scheme had the defect of allowing a small number of filaments, or even a single fixed filament, to sterically inhibit bacterial motion in a given direction. Exploring bipolar ActA distributions, or distributions with ActA anywhere near the forward tip of the bacterium, was therefore not possible. Filaments now accumulate “drag units” stochastically as a function of their length to more realistically capture the implicit interaction of filaments with each other and with other cellular entities (their surroundings). The more “drag units” a filament has, the greater its viscous drag coefficient that multiplies its velocity relative to the lab frame to compute the viscous drag force resisting its motion. While the drag coefficient of an individual filament/branch can grow large, it can always be moved out of the way with sufficient force.

#### Amontons' law for filament-bacteria interactions

The simulation detects collisions between actin filaments and the bacterium at each time-step, and these collisions are resolved through application of equal and opposite forces. . Let 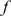 be the vector force a filament tip exerts on the bacterium. In general we can resolve this into one component normal to the bacteria surface plus another tangent to the bacterial surface. For a perfectly smooth bacterium [as in 12] the normal component, 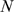, is the only non-zero component. A more realistic bacterial surface is characterized by a non-zero friction coefficient 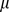, and therefore a friction force tangent to the bacterial surface with magnitude 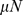 and direction opposite the instantaneous bacterial velocity. This is Amontons' law, familiar to us in the macroscopic world. Typical engineering materials have coefficients of kinetic friction in the range of 0.005 to 0.2 –the coefficient of friction for actin filaments on gram-positive bacterial surfaces is not known. Discussion of Amontons' law at the microscopic scale can be found in [28]–[30].

#### Bacterial drag

The bacterium was given a constant shape-based drag in Alberts and Odell [12]. The exact nature of the mechanisms that restrain the bacterium is the main focus of this manuscript. We extensively study the effect of ActA – f-actin tether properties, fluid coupling between the bacterium and its surrounding network of actin filaments, and friction between filaments and the bacterial surface (Fig. S2).

#### ActA-filament interaction probability

The probability that an actin filament encountering the bacterial surface will interact with an ActA protein is now dependent on the concentration of ActA proteins at that location on the bacterium (the concentration depends only on the arc length measured from the posterior pole – not on the azimuthal angle). We previously assumed that all filaments colliding with the bacterium would immediately interact with an ActA protein – a good approximation in regions of high ActA density, which is distribution dependent – but no longer relevant when ActA concentration tapers off gradually towards the front end of the bacterium.

#### Random number generation

Random number generation is too important to be left to chance. Instead of using the built-in Java pseudo-random number generator (period of 2^48^−1), we now employ the scientific standard Mersienne Twister method (period of 2^19937^−1). This is implemented from the free and open-source Java package provided by www.honeylocust.com/RngPack/.

## Supporting Information

Text S1 Description of the agent-based model.
(0.08 MB DOC)

Text S2 Derivation and solution of the continuum model.
(0.21 MB DOC)

Text S3 Mathematica notebook solution of discretized continuum model.
(0.13 MB PDF)

Figure S1 Snapshots of *in silico L. monocytogenes* motility (adapted from Alberts and Odell 2004). A) A bacterium with a normal ActA distribution (Fig. 1B) moves at speeds similar to experimental measurements with a realistically dense actin tail. B) An illustration detailing the states considered in the simulation. Filaments can branch from existing filaments, become capped at their barbed-ends by Capping protein, bind ActA proteins on the bacterial surface, collide with the bacterium to produce propulsive force, etc. Hydrolysis of filamentous actin monomers is assumed to be fuse-like, i.e. there are distinct regions of ATP, ADP-Pi, and ADP actin, as opposed to a more likely random hydrolysis and dissociation for each monomer.
(0.85 MB EPS)

Figure S2 Of the restraining forces considered in the agent-based model, only the frictional force between filaments and the bacterial surface is cooperative. A) The top bacterium has three transient ActA-filament tethers along the side of the bacterium, while the bottom one has six. A two-fold increase in tethers will, on average, restrain the bacterium with only twice the force, i.e. restraint by ActA-filament tethers is approximately linear in the number of interacting filaments. B) At low Reynolds number the induced fluid velocity field can extend a large distance from an object's surface. The top figure shows a hypothetical fluid flow profile -vf will equal the bacterial velocity v at the surface and decrease parabolically from there. Any nearby filaments will interact with this fluid and induce a drag on the bacterium in a complicated way, dependent on their own position, orientation and velocity. We approximate this interaction by simply counting the number of filaments within a ‘shell’ about the bacterium and increase the drag coefficients of the bacterium linearly with that number. The induced drag will increase linearly, at most, as demonstrated in the bottom figure. Filaments i and ii will independently interact with the bacterium's fluid velocity field; identically positioned, oriented, and moving filaments will contribute equally to the drag on the bacterium, i.e. the drag will increase approximately linearly with the number of filaments. A filament pair as in iii, however, will effectively appear as a single filament in this fluid coupling -at high filament densities we might expect the drag to increase even less than linearly with the number of filaments. C) By Amonton's law, kinetic friction is proportional to the normal contact force, as shown in the top figure. The bottom figure illustrates how many filaments can cooperate to increase the average normal force, i.e. N∝β where n is the number of contributing filaments and β is an unknown exponent of dependence. The total friction force is just a summation of the contribution from each of the n filaments, and thus is cooperative in n by the factor β, i.e. F_Total_∝n^1+β^. In Fig. S3 we determine average values for β in the agent-based simulation.
(0.71 MB EPS)

Figure S3 Examination and determination of continuum model parameters through analysis of average agent-based model relationships. A) Averaging of data for three *in silico* bacteria with our standard ultrapolar ActA distribution (Fig. 1B) including kinetic friction. Autocatalytic barbed-end creation rate, propulsive force on the rear hemisphere of the bacterium, and total radial contact force for filaments on the cylindrical section of the bacterium are averaged over 0.1, 1, and 10 sequential seconds. Only over intervals of 10 seconds or more (third row) does an assumption of a constant average value seem reasonably valid. These data also demonstrate the general way that nanoscale agent-based model might be used to inform parameter choices for a microscale continuum model. For example, the slope of the best linear relation between propulsive force and number of barbed-ends at the rear of the bacterium is the propulsive force per filament, while the exponent of the power relationship between contact force and filament tips at the surface defines our cooperativity factor β. B) A larger dataset of 10 *in silico* bacteria each for ultrapolar (blue) and normal (red) ActA profiles (Fig. 1B) reveals that the average autocatalytic barbed-end creation rate, propulsive force per filament, and even the cooperativity factor β are functions of ActA distribution. Each datapoint is the average of 1000 sorted 0.1 second sequential averages, e.g. we group 0.1 second averages of total propulsive force by the number of filaments on the rear hemisphere of the bacterium, then average 1000 adjacent values. These data suggest (for normal bacteria) values of 0.07 new barbed-ends for each existing barbed-end per second, 0.07 pN per filament tip at the rear hemisphere, and a cooperativity factor β = 0.65.
(20.39 MB EPS)

Figure S4 We devised a simple function, the sum of two sine waves (i. e. the first two terms in a Fourier series approximation), to distribute ActA on our *in silico* bacteria. By varying a single parameter, A, we can span the range of ActA 1st moments measured in our experimental data set and reasonably represent the typical shapes of those distributions. For comparison, our prototypical normal (red) and ultrapolar (blue) experimentally-measured distributions are shown alongside these function-generated distributions.
(0.78 MB EPS)

Figure S5 Comparison of *in silico*, and experimental actin and distribution. The top row shows an average experimental actin distribution along the bacterium for an ultrapolar and normal ActA bacterium. Linescans from four timepoints in a timelapse movie were averaged to obtain these plots. The bottom row shows an *in silico* average actin distribution along the bacterium obtained from our simulations. Actin distributions along the bacterium were averaged over the course of 30 seconds in the simulation. Comparing the *in silico* and experimental actin profiles reveals that they share distinctive features for both normal and ultrapolar ActA distributions.
(0.92 MB EPS)

Table S1 Concentrations used in the agent-based model (reproduced from Alberts and Odell 2004). These values are not for any specific cell type, but are typical biological concentrations and similar to those used for *in vitro* reconstitutions of bacterial motility.
(0.02 MB DOC)

Table S2 Rates used in the agent-based model (reproduced from Alberts and Odell 2004). A hydrolysis rate is given for a vectorial ATP hydrolysis model; experimental evidence currently supports the random hydrolysis model but we have, for simplicity, implemented a vectorial scheme for this analysis. That is, we assume that there is a distinct border within each filament between the ATP actin, ADP-Pi actin, and ADP actin regions; only monomers adjacent to these borders can transition from ATP actin to ADP-Pi actin or from ADP-Pi actin to ADP actin. We can readily switch to a random hydrolysis model in future studies. The values in angle brackets, for the interactions between ActA, Arp2/3, and actin monomers, are calculated considering the diffusive flux onto the bacterium's surface (see Alberts and Odell, 2004:Dataset S2). These values are thus dependent upon ActA density, the concentrations of Arp2/3 and actin, and a heuristic adjustment of these rates to balance new filament nucleation and side-branching in order to achieve realistic tail morphologies. The on-rates in brackets listed here apply to the concentrations in Table S1.
(0.03 MB DOC)

Video S1 Example trajectories of normal (on the left) and ultrapolar (on the right) *in silico* bacteria with friction between filaments and the bacterial surface. Only regions of relatively smooth motion are analyzed to determine the average speed of such bacteria, i.e large stalls and hops are ignored.
(5.58 MB MOV)
