# Supplementary material for: An Experimental and Computational Study of the Effect of ActA Polarity on the Speed of Listeria monocytogenes Actin-based Motility
Source: PLoS Comput Biol. 2009 Jul 10;5(7):e1000434. doi: 10.1371/journal.pcbi.1000434 (PMC2699634; doi:10.1371/journal.pcbi.1000434)
Supplement: Text S1 — Description of the agent-based model. (0.08 MB DOC) [file pcbi.1000434.s001.doc]

**Text S1: Description of the agent-based *in silico* model and parameter selection**

# 1. Model Description

The *in silico* model used in this study is largely as described in [1]. Using the object-oriented Java language, we created an agent-based simulation in which each individual actin filament is represented as a rigid cylinder. Monte Carlo simulations of biochemical events --polymerization, capping, and f-actin incorporated ATP hydrolysis—are used to elongate, shrink, and otherwise change the biochemical state of these filaments. Filaments can become elements of larger structures, as shown in the dendritic branch in Fig. S1B, which then move together as a rigid body. Interaction of f-actin barbed-ends with ActA proteins on the bacterial surface catalyze two important biochemical events: barbed-end uncapping (if capped) and the binding of an ActA-bound Arp2/3 at the attachment point, leading to a side-branching daughter filament (in the model a side-branch only forms from the trimeric complex of ActA-filament-Arp2/3). Filament-ActA bonds are elastic tethers, generally working keep the bacterium close to the actin tail and specific f-actin barbed-ends. These tethers break in a strain-dependent manner.

A simulation typically begins without any filaments. Filaments are nucleated de novo by explicitly represented ActA proteins on the bacterium. Note that this nucleation might actually represent the capture of short cytoplasmic actin filaments. The specified ActA distribution on the bacterial surface determines the overall rate of nucleation, and the availability of ActA proteins for capture by existing barbed-ends, at any location on the bacterium. The length-wise asymmetry of the ActA distribution precludes any symmetry breaking problem for the bacterium. The initial f-actin cloud about the bacterium is soon sufficient to move the bacterium, and motility begins. An actin filament is released from an ActA if the mechanical tether between the two is overly-strained, or if an Arp2/3 mediated side-branching event occurs. These filaments might then be capped, interact with the same or other ActA proteins, or both.

# *What the model gets mostly right*

## Biochemical rates

Many of the critical biochemical rates in the simulation, such as those for polymerization and capping, are taken from measurements in the literature (Table S1 and S2). The frequency of Arp2/3 mediated side-branching is a function of many model parameters –we can adjust unknown rates to match the f-actin density from our fluorescent measurements, as discussed in Parameter Selection.

## Force-based interactions

Newton’s laws govern the force-based interactions between filaments and the bacterium, thus the mechanics are valid to the extent that each body’s resistance to motion (be it viscous or elastic) is appropriately represented. The model captures the geometries of the biologic system, e.g. the curvature of the bacterial surface and the filament orientations in a dendritic actin network. Geometry is important in determining the direction of force for each filament-bacterium force interaction.

## Position and force affect biochemical events

In the model, the polymerization and capping rates for a given filament are modulated by the filament’s position (i.e. steric accessibility of the tip to monomers and capping protein). Likewise, Arp2/3 binding to the sides of filaments depends on interaction with ActA, and the availability of a binding site (i.e. each Arp2/3 occludes others from binding in the same location). Our model encodes these important details in the interplay between biochemical and force-based interactions.

# *Limitations of the current model*

## Gel effects

The filaments and filament branches in the model don’t interact with one another through either crosslinkers or entanglements. Instead the viscous drag of a filament increases over time as a function of length. In this way older actin filaments and structures are progressively fixed in space. For this reason, the model doesn’t directly represent any gel effects. Flexible filaments that collide and interact with one another through crosslinkers would vastly improve the model in this regard. Detecting collisions between the many rigid filament segments that might be used to appropriately capture the flexibility of many thousands of actin filaments, and resolving the many associated force interactions, represents a significant increase in computational burden over the existing model. But such a model is increasingly appealing, and attainable, with gains in computational power.

## Hydrolysis and depolymerization

The model presently uses a fuse-like model for hydrolysis –there is a distinct region of ATP, ADP-Pi, and ADP in each filament (Table S2). Depolymerization is achieved by increasing the pointed-end depolymerization rates, rather than through explicit cofilin severing. This scheme is computationally advantages as cofilin severing would make many filaments out of one, increasing the number of agents to track. Given the steady-state speed of bacteria, network depolymerization largely occurs far from the bacterial surface. Therefore, this flawed representation of depolymerization does not impact answers to the questions we pose in this work. Nontheless, simulation of this process would be improved by considering the random hydrolysis, dissociation, and cofilin binding for each monomer, and a set of explicit cofilin severing rules.

## Explicit ActA proteins

New work [2] suggests that the ActA surface density estimate we used in this work (see Table S1) is an order of magnitude too large. Currently, a population of ActA proteins (4000 – 10,000) are explicitly simulated as nucleators, and new explicit ActAs are created as needed to interact with colliding barbed-ends. I.e. there is a large population of ActA proteins that is not explicitly represented until needed for a particular force / biochemical interaction with actin filaments. The new lower estimate for the number of ActA proteins on a single bacterium (~5000) would allow us to explicitly simulate each individual ActA.

## Fluid interactions

The exchange of forces between nearby bodies in a viscous fluid is difficult to simulate, at best. We largely ignore this fluid coupling and calculate the viscous drag on densely-packed bodies as though each body were in isolation. When implementing a fluid coupling between filaments and the bacterium, however, we use the formula given in Supplemental Text S2, Eq 6.

# 2. Parameter Selection

Many of the fundamental parameters that govern model behavior are directly specified or constrained by experimental measurement. Others are informed by coarse physics approximations. Still others can only be indirectly ‘tuned’ to match some larger scale observable, such speed of motion or density of the actin tail. Here we lay out our rationale in parameter selection.

### Directly measured rates

Wherever possible we have taken parameter values directly as reported in the literature. Tables S1 and S2 are reproduced from [1] showing experimentally determined concentrations and rates, respectively.

# *Tuning to macro-observables*

The more difficult task lay with setting the unknown parameter values informed by neither experimental measurement or physics approximation. These parameters may encode mechanistic details (e.g. characteristics of filament-ActA tethers and maximal ActA binding density on a filament) or biochemical rates, such as the time-scale for side-branch creation by an ActA-bound filament. This difficulty is tempered by the inherent robustness of cellular behaviors, and thus the complex models that mimic them. In other words, the essential emergent behaviors in our Listeria motility model should not be fragile with respect to the aforementioned unknown parameters. Nonetheless, we can tune some unknown parameters so that larger-scale *in silico* properties closely match their experimental counterparts.

*Actin tail characteristics:*

The length and f-actin density in the tails of motile bacteria can be estimated from the fluorescently labeled actin signal in our experiments. Order of magnitude estimates are appropriate here, since these features are largely dependent on ActA intensity and distribution, actin concentration, etc. In the model we thus ‘tuned’ two parameters --de novo f-actin nucleation and the side-branching probability for an f-actin—ActA bond—to generate highly branched and dense tails with on the order of one hundred filaments about the spherical hemisphere, hundreds of filaments interacting with the bacterial surface all together, and thousands of filaments within the first few bacterial lengths of the tail.

Beyond these coarse targets for numbers of filaments, the *in silico* distribution of actin density along the bacterium can be compared with fluorescent images. Fig. S5 shows the reasonable match between the shape of the *in silico* and experimental actin distributions. Additionally, we can experimentally estimate the number of actin monomers from our fluorescent signal and compare with direct monomer counts from the simulation. We find that our best estimate of experimental actin is a very close match with our simulations (within 10%). Considering all possible errors in measuring actin concentration, degree of fluorescent labeling, and accuracy of sequential dilutions our estimates are at worst different by 2.5-5-fold (data not shown).

*Bacterial speed:*

We have less direct control over bacterial speed than might naively be assumed. There is no single parameter that we can dial up/down to increase/decrease the average speed at which the *in silico* bacterium moves. For instance, speed does not vary linearly with assumed viscosity --greater resistance to bacterial motion feeds back onto the biochemical interactions to create denser, higher propulsive force networks. Bacterial speed can, in fact, be considered an emergent behavior of the complex underlying interaction. We use reasonable bacterial speed (40 – 150 nm/s) as an additional target macro-observable in our tuning of nucleation and branching rates, and fluid coupling between the bacterium and the surrounding filaments.

**3. Flux of actin monomers to the bacterium is not limiting**

Here we justify our model assumption that actin polymerization near the bacterial surface is not limited by diffusion of actin monomers. The transport rate of a protein to the surface of a sphere can be found from the Heat Equation as

(1)

where is the diffusivity of the protein, is the sphere’s radius, and and are the protein concentrations far from the sphere and at the sphere’s surface, respectively. Applying Dirichlet boundary conditions, we let be constant at 12µM and set . G-actin has a mass of about 42 kD and a radius of 2.7 nm. Using the Stokes equation for the drag coefficient of a sphere, , and the Einstein equation we can estimate the diffusivity of G-actin.

(2)

where is the viscous drag, is the fluid viscosity (we use 3.0 mPa-s [3]), is Boltzmann’s constant, is the temperature in Kelvin, and is the diffusivity. This diffusivity doesn’t account for the crowded, inhomogenous environment of the cell. In consideration of the work of Luby-Phelps ([4,5]) we modify this “nominal” diffusivity by heuristically fitting a curve to experimental data (Fig. 3 in [5]):

(3)

with in nanometers. For an actin monomer, we find and thus . Using this value in our transport equation, we find a G-actin flux onto a sphere of radius 0.35µm of

(4)

An alternative choice for actin monomer diffusivity, a measurement with ActA generated actin gels, comes from [6]. Using their value of , we estimate a 6-fold lower G-actin flux of ~63,000 monomers/second. We might halve this number to consider diffusion to only one hemisphere of the bacterium --the forward section of the bacterium occludes diffusion to the tail. Nevertheless, these estimates suggest that between 400 - 2,500 filaments at the bacterial surface could elongate at 200 nm/s, the higher end of bacterial speeds in our experiments and simulations. There are seldom more than 250 filaments interacting with the rear hemisphere of in our simulations, and presumably in the biology (data not shown). Thus, we don’t find actin monomer diffusion limiting.

**References:**

1. Alberts JB, Odell GM (2004) In silico reconstitution of Listeria propulsion exhibits nano-saltation. PLoS Biol 2: e412.

2. Footer MJ, Lyo JK, Theriot JA (2008) Close Packing of Listeria monocytogenes ActA, a Natively Unfolded Protein, Enhances F-actin Assembly without Dimerization. J Biol Chem 283: 23852-23862.

3. Dembo M (1989) Mechanics and control of the cytoskeleton in Amoeba proteus. Biophys J 55: 1053-1080.

4. Luby-Phelps K (1994) Physical properties of cytoplasm. Curr Opin Cell Biol 6: 3-9.

5. Luby-Phelps K (2000) Cytoarchitecture and physical properties of cytoplasm: volume, viscosity, diffusion, intracellular surface area. Int Rev Cytol 192: 189-221.

6. Plastino J, Lelidis I, Prost J, Sykes C (2004) The effect of diffusion, depolymerization and nucleation promoting factors on actin gel growth. Eur Biophys J 33: 310-320.
