# Supplementary material for: An Experimental and Computational Study of the Effect of ActA Polarity on the Speed of Listeria monocytogenes Actin-based Motility
Source: PLoS Comput Biol. 2009 Jul 10;5(7):e1000434. doi: 10.1371/journal.pcbi.1000434 (PMC2699634; doi:10.1371/journal.pcbi.1000434)
Supplement: Text S2 — Derivation and solution of the continuum model. (0.21 MB DOC) [file pcbi.1000434.s002.doc]

**Text S2: A partial-differential equation model of *Listeria* motility**

This simple model directly relates motion of a bacterium to the actin population it creates about itself. A simplistic formulation, but one that leaves out several dynamic subsystems included in the complex stochastic model, tracks three state variables, the number of barbed-ends (), the amount of actin (), and the velocity of the bacterium (), through time and across the one-dimensional space of the bacterium. Refer to Fig. 5, which shows a spatially discretized schematic of the geometry considered here.

# Formulation

We consider a one-dimensional coordinate system moving with the bacterium. The derivation of the governing equations involves two conservation laws for scalar fields in one-dimensional space, namely the densities of barbed-ends and actin filaments. This universal conservation law (or continuity equation) for a scalar field, , is

(1)

where is the flux density of . In our formulation neither nor moves by diffusion, so the flux density of those quantities, seen from a coordinate system attached to the bacterium, are and , respectively. Thus the standard convective (or Stokes) derivative, to which we add creation rate density terms

(2)

(3)

(4)

where and are Stokes time-derivatives. and represent the autocatalytic and *de novo* creation rate densities of new barbed-ends, respectively, and is the creation rate density of polymerized actin --a function of existing barbed-ends. At low Reynolds number, the momentum balance equation for an object is that the viscous drag (proportional to velocity) and applied forces sum to zero (no inertial effects). Thus the velocity for an object is just a ratio of applied force (a function of barbed-ends) to drag coefficient (a function of total actin).

We are left only with a decision on the exact form for and . To be consistent with decisions made in the complex model, we assume that barbed-ends generate a propulsion force proportional to the dot product of local normal of the bacterial surface and the direction of motion, i.e. a propulsion force in the x-direction is only produced by barbed-tips near the hemispherical caps of the bacterium, with decreasing contribution as we move from tip to the central cylinder. Mathematically

(5)

where is a function that defines the cosine of the bacterial surface normal with the x-direction as we go from tip-to-tip. The drag coefficient for the bacterium is likewise calculated as an integral over the length of the bug, the exact form depends on what restraining mechanism is being considered. With Stokes (laminar) flow, we expect the drag contributions from each actin filament will simply superpose, i.e. we expect the bacterial drag to increase linearly (at most, Fig. S2) with the number of filaments about the bacterium. We sum actin (possibly with a weighting factor) and put that sum into a linear function for bacterial drag

(6)

where is a shape-based value for the drag (if there were no surrounding actin network), multiplies that shape-based drag in consideration of cytoplasmic properties, and defines the average increase in bacterial drag per actin filament. We can estimate from the literature [1], and from the theoretical increase in transverse drag on a filament near a plane [2]. The actin filaments that contribute to this fluid drag between filaments and the bacterium, , are summed as

(7)

where (l) is a weighting factor that allows us to modify the relative drag contribution from actin on the hemispherical caps or the cylindrical sides of the bacterium, although in our solution we let for all .

For restraint by ActA-filament tethers or by kinetic friction between filaments and the bacterial surface the drag term becomes a function of , the number of barbed-ends. I.e.

(8)

where is the cooperativity of the restraining mechanism (zero for ActA-filament tethers), is the average drag increase per barbed-end, and is an integral summation of barbed-ends in the form of equation 7.

# Solution

To solve these non-linear partial-differential equations numerically, we have chosen to spatially discretize along the bacterial length with spacing (Fig. 5A) to convert the two PDEs into a system of coupled ODEs, which we solve by a standard numerical algorithms. Note that we use “upwind” differencing (not centered differencing) to discretize the convective term. What follows is a synopsis of our solution approach in the supplemental Mathematica notebook.

With state variables , representing barbed-ends, filamentous actin, and bacterial position, we have 17 differential equations each for and (one for each element ). Let and represent the time-derivatives of the number of barbed-end and amount of actin, respectively, then

(9)

(10)

where , , and are the coefficients for barbed-end autocatalytic increase, barbed-end de novo creation, and filamentous actin growth, respectively. One final differential equation, as in equation 4, for bacterial position is

(11)

In our discretization, only the first four mesh elements contribute to the forward propulsion of the bacterium. Each barbed-end in these elements is assumed to contact the bacterial surface with force . The total propulsive force is taken as the sum of these forces, weighted by the average dot product of the surface normal to direction of motion for each element, i.e. equation 5 in our actual solution becomes

(12)

where is the number of barbed-ends in element *i*. All elements are assumed to contribute, via fluid coupling, to the drag on the bacterium of length and radius

(13)

where is an approximation for the shape-based drag for a pill-shaped bacteria in fluid with viscosity , is the base increase over the shape-based drag considering the generic properties of cytoplasm, is just the summation of all filaments about the bacterium, and is an average effect of an actin filament on bacterial drag.

To model ActA-filament tethers or kinetic friction we reformulate the drag in terms of number of barbed-ends, as in equation 8. The cooperativity for tethers while the agent-based model suggests a between 0.6 and 0.7 for kinetic friction (Fig S3).

A solution using these discretized equations and the values reported below is shown in the attached Mathematica notebook --this code is easily modified (by anyone with a Mathematica license) to explore the effect of our model assumptions and parameter values.

# Parameter values

Values shown with an asterisk (*) can be freely varied in the model. Values with a pound (#) are justified by evaluation of agent-based emergent properties.

Bacterial length µm

Bacterial radius µm

Viscosity Pa-s [3]

Spatial discretization µm

Barbed-end autocatalytic creation factor #

Barbed-end de novo creation factor : depends on ActA intensity in each element *

F-actin growth as a function of barbed-ends *

Shape-based drag [2]

Cytoplasmic properties increase shape-based drag [1]

F-actin dependent increase over shape-based drag *

Propulsive force per actin filament #

Cooperativity of restraining mechanism = 0.6 – 0.7 #

Fig. S3B shows how an analysis of the agent-based model can be used to establish average relationships between observables that are not decreed, but rather emerge from the encoded nanoscale interactions. The values we report in Fig. S3B, for propulsive force per actin filament on the rear hemisphere of the bacterium and for the barbed-end autocatalytic creation rate, justify the values we’ve used in the continuum model.

**References:**

1. Giardini PA, Theriot JA (2001) Effects of intermediate filaments on actin-based motility of Listeria monocytogenes. Biophys J 81: 3193-3203.

2. Howard J (2001) Mechanics of Motor Proteins and the Cytoskeleton. Sunderland, MA: Sinauer Associates.

3. Dembo M (1989) Mechanics and control of the cytoskeleton in Amoeba proteus. Biophys J 55: 1053-1080.
