# Supplementary material for: An Experimental and Computational Study of the Effect of ActA Polarity on the Speed of Listeria monocytogenes Actin-based Motility
Source: PLoS Comput Biol. 2009 Jul 10;5(7):e1000434. doi: 10.1371/journal.pcbi.1000434 (PMC2699634; doi:10.1371/journal.pcbi.1000434)
Supplement: Text S3 — Mathematica notebook solution of discretized continuum model. (0.13 MB PDF) [file pcbi.1000434.s003.pdf]

Text S3 : Solution to a continuum model for Listeria motility
A supplement for Rafelski, Alberts, and Odell 2009.
Refer to Figure 5 for model schematic.

Parameter Values (Text S2)

In[1284]:=
```
bugLength = 1.7; (* microns *)
R = 0.35; (* microns... the radius of the spherical cap and cylinder *)
d = 0.1; (* microns.... the mesh size *)
elementNum = bugLength / d; (* number of mesh elements... make sure this is an integer *)

aeta = 0.003; (* Pa-s ... 0.001 Pa-s = 1 centipoise = viscosity of water *)
gammaShape = (4 Pi aeta bugLength / 2) / (Log[bugLength / R] - .5);
(* shape-based drag for a pill-shaped bacterium *)
baseV = 25; (* the base shape-based drag multiplier... added to by the function below *)
rCoeff = 0.6; (* the constant multiplier in the drag function *)
beta = 0.0; (* the cooperativity of the restraining mechanism... use 0 for linear *)
shapeX = baseV + rCoeff x^(1 + beta);
(* the value of shapeX from this function is the multiplier of the shape-based drag for the bacterium *)

autoC = .1; (* autocatalytic barbed-end creation *)
CsubF = .1; (* pN/filament ... the force generated per filament in BTail *)
polyC = 3.4; (* actin growth as function of barbed-ends --not calibrated to any actual actin measure *)
simTime = 480; (* simulation time to run out to... in seconds*)
```

In[2]:=
ActA Distribution Options (Fig 1 B)

In[1286]:=
```
nucNM = Table[{0.038, 0.064, 0.084, 0.093, 0.091, 0.090, 0.086,
        0.080, 0.080, 0.074, 0.064, 0.057, 0.044, 0.032, 0.018, 0.006, 0.000}]; (* std norm *)
nucUP = Table[{0.074, 0.109, 0.119, 0.108, 0.092, 0.077, 0.067, 0.061, 0.058, 0.053,
        0.050, 0.044, 0.037, 0.029, 0.017, 0.007, 0.000}]; (* std ultrapolar *)
ListPlot[{nucNM, nucUP}, Joined → True, PlotRange → {{1, elementNum}}, Axes → {True, False},
     AxesLabel → {"bin", "nucleator distribution"}]; (* remove semi-colon to see plot! *)
nuc = nucNM; (* set which of the above distributions you want to use here *)
```

Propulsive Force and Choice of Restraining Mechanism

In[1289]:=
```
Clear[F, gammma, maxNuc];
ForceElements = 0.857 B1[t] + 0.571 B2[t] + 0.286 B3[t] + 0.036 B4[t];
(* only the first 4 elements can propel the bug,
weighted by average dot product of surface normal and path direction *)
DragElementsActin = N1[t] + N2[t] + N3[t] + N4[t] + N5[t] + N6[t] + N7[t] + N8[t] + N9[t] + N10[t] +
        N11[t] + N12[t] + N13[t] + N14[t] + N15[t] + N16[t] + N17[t] ; (* all actin elements contribute *)
DragElementsTethers = B1[t] + B2[t] + B3[t] + B4[t] + B5[t] + B6[t] + B7[t] + B8[t] +
        B9[t] + B10[t] + B11[t] + B12[t] + B13[t] + B14[t] + B15[t] + B16[t] + B17[t];
DragElementsFriction = B4[t] + B5[t] + B6[t] + B7[t] + B8[t] + B9[t] + B10[t] +
        B11[t] + B12[t] + B13[t] + B14[t] + B15[t] + B16[t] + B17[t];
F = CsubF ForceElements; (* force proportional to the number of filaments in the tail cap *)

(* for Fluid Coupling use DragElementsActin below, rCoeff = 0.6, and beta = 0 *)
(* for ActA-Filament Tethers use DragElementsTethers below, rCoeff = 8.0, beta = 0 *)
(* for Kinetic Friction use DragElementsFriction below, rCoeff = 5.0, beta = 0.2 *)
gamma = ( shapeX /. x → DragElementsActin) gammaShape; (* gamma is the bug's drag *)
```

The ODEs –– one for velocity of the bacterium, 17 each for barbed – ends (the bEqs) and actin (the nEqs)

In[1296]:=

```
posEq = x'[t] == F / gamma;  (* just v = F/gamma *)
posEqInit = x[0] == 0.0;  (* initial condition on position... set to zero at t=0 *)

b1Eq = B1'[t] == maxNuc nuc[[1]] (2 Pi R d) + autoC B1[t] + (B2[t] / d – B1[t] / d) x'[t];
b1EqInit = B1[0] == 0;
n1Eq = N1'[t] == polyC B1[t] + (N2[t] / d – N1[t] / d) x'[t];
n1EqInit = N1[0] == 0;

b2Eq = B2'[t] == maxNuc nuc[[2]] (2 Pi R d) + autoC B2[t] + (B3[t] / d – B2[t] / d) x'[t];
b2EqInit = B2[0] == 0;
n2Eq = N2'[t] == polyC B2[t] + (N3[t] / d – N2[t] / d) x'[t];
n2EqInit = N2[0] == 0;

b3Eq = B3'[t] == maxNuc nuc[[3]] (2 Pi R d) + autoC B3[t] + (B4[t] / d – B3[t] / d) x'[t];
b3EqInit = B3[0] == 0;
n3Eq = N3'[t] == polyC B3[t] + (N4[t] / d – N3[t] / d) x'[t];
n3EqInit = N3[0] == 0;

b4Eq = B4'[t] == maxNuc nuc[[4]] (2 Pi R d) + autoC B4[t] + (B5[t] / d – B4[t] / d) x'[t];
b4EqInit = B4[0] == 0;
n4Eq = N4'[t] == polyC B4[t] + (N5[t] / d – N4[t] / d) x'[t];
n4EqInit = N4[0] == 0;

b5Eq = B5'[t] == maxNuc nuc[[5]] (2 Pi R d) + autoC B5[t] + (B6[t] / d – B5[t] / d) x'[t];
b5EqInit = B5[0] == 0;
n5Eq = N5'[t] == polyC B5[t] + (N6[t] / d – N5[t] / d) x'[t];
n5EqInit = N5[0] == 0;

b6Eq = B6'[t] == maxNuc nuc[[6]] (2 Pi R d) + autoC B6[t] + (B7[t] / d – B6[t] / d) x'[t];
b6EqInit = B6[0] == 0;
n6Eq = N6'[t] == polyC B6[t] + (N7[t] / d – N6[t] / d) x'[t];
n6EqInit = N6[0] == 0;

b7Eq = B7'[t] == maxNuc nuc[[7]] (2 Pi R d) + autoC B7[t] + (B8[t] / d – B7[t] / d) x'[t];
b7EqInit = B7[0] == 0;
n7Eq = N7'[t] == polyC B7[t] + (N8[t] / d – N7[t] / d) x'[t];
n7EqInit = N7[0] == 0;

b8Eq = B8'[t] == maxNuc nuc[[8]] (2 Pi R d) + autoC B8[t] + (B9[t] / d – B8[t] / d) x'[t];
b8EqInit = B8[0] == 0;
n8Eq = N8'[t] == polyC B8[t] + (N9[t] / d – N8[t] / d) x'[t];
n8EqInit = N8[0] == 0;

b9Eq = B9'[t] == maxNuc nuc[[9]] (2 Pi R d) + autoC B9[t] + (B10[t] / d – B9[t] / d) x'[t];
b9EqInit = B9[0] == 0;
n9Eq = N9'[t] == polyC B9[t] + (N10[t] / d – N9[t] / d) x'[t];
n9EqInit = N9[0] == 0;

b10Eq = B10'[t] == maxNuc nuc[[10]] (2 Pi R d) + autoC B10[t] + (B11[t] / d – B10[t] / d) x'[t];
b10EqInit = B10[0] == 0;
n10Eq = N10'[t] == polyC B10[t] + (N11[t] / d – N10[t] / d) x'[t];
n10EqInit = N10[0] == 0;

b11Eq = B11'[t] == maxNuc nuc[[11]] (2 Pi R d) + autoC B11[t] + (B12[t] / d – B11[t] / d) x'[t];
```

```
b11EqInit = B11[0] == 0;
n11Eq = N11'[t] == polyC B11[t] + (N12[t] / d – N11[t] / d) x'[t];
n11EqInit = N11[0] == 0;

b12Eq = B12'[t] == maxNuc nuc[[12]] (2 Pi R d) + autoC B12[t] + (B13[t] / d – B12[t] / d) x'[t];
b12EqInit = B12[0] == 0;
n12Eq = N12'[t] == polyC B12[t] + (N13[t] / d – N12[t] / d) x'[t];
n12EqInit = N12[0] == 0;

b13Eq = B13'[t] == maxNuc nuc[[13]] (2 Pi R d) + autoC B13[t] + (B14[t] / d – B13[t] / d) x'[t];
b13EqInit = B13[0] == 0;
n13Eq = N13'[t] == polyC B13[t] + (N14[t] / d – N13[t] / d) x'[t];
n13EqInit = N13[0] == 0;

b14Eq = B14'[t] == maxNuc nuc[[14]] (2 Pi R d) + autoC B14[t] + (B15[t] / d – B14[t] / d) x'[t];
b14EqInit = B14[0] == 0;
n14Eq = N14'[t] == polyC B14[t] + (N15[t] / d – N14[t] / d) x'[t];
n14EqInit = N14[0] == 0;

b15Eq = B15'[t] == maxNuc nuc[[15]] (2 Pi R d) + autoC B15[t] + (B16[t] / d – B15[t] / d) x'[t];
b15EqInit = B15[0] == 0;
n15Eq = N15'[t] == polyC B15[t] + (N16[t] / d – N15[t] / d) x'[t];
n15EqInit = N15[0] == 0;

b16Eq = B16'[t] == maxNuc nuc[[16]] (2 Pi R d) + autoC B16[t] + (B17[t] / d – B16[t] / d) x'[t];
b16EqInit = B16[0] == 0;
n16Eq = N16'[t] == polyC B16[t] + (N17[t] / d – N16[t] / d) x'[t];
n16EqInit = N16[0] == 0;

b17Eq = B17'[t] == maxNuc nuc[[17]] (2 Pi R d) + autoC B17[t] – (B17[t] / d) x'[t];
b17EqInit = B17[0] == 0;
n17Eq = N17'[t] == polyC B17[t] + (N17[t] / d) x'[t];
n17EqInit = N17[0] == 0;
```

Speed as a Function of De Novo Barbed – end Nucleation Rate

```
In[1364]:= Speeds = Table[
      posAns = NDSolve[{posEq, b1Eq, b2Eq, b3Eq, b4Eq, b5Eq, b6Eq, b7Eq, b8Eq, b9Eq, b10Eq, b11Eq, b12Eq,
          b13Eq, b14Eq, b15Eq, b16Eq, b17Eq, posEqInit, b1EqInit, b2EqInit, b3EqInit, b4EqInit, b5EqInit, b6EqInit,
          b7EqInit, b8EqInit, b9EqInit, b10EqInit, b11EqInit, b12EqInit, b13EqInit, b14EqInit, b15EqInit, b16EqInit,
          b17EqInit, n1Eq, n2Eq, n3Eq, n4Eq, n5Eq, n6Eq, n7Eq, n8Eq, n9Eq, n10Eq, n11Eq, n12Eq, n13Eq,
          n14Eq, n15Eq, n16Eq, n17Eq, n1EqInit, n2EqInit, n3EqInit, n4EqInit, n5EqInit, n6EqInit, n7EqInit,
          n8EqInit, n9EqInit, n10EqInit, n11EqInit, n12EqInit, n13EqInit, n14EqInit, n15EqInit, n16EqInit, n17EqInit},
         {x[t], B1[t], B2[t], B3[t], B4[t], B5[t], B6[t], B7[t], B8[t], B9[t], B10[t], B11[t], B12[t], B13[t],
          B14[t], B15[t], B16[t], B17[t], N1[t], N2[t], N3[t], N4[t], N5[t], N6[t], N7[t], N8[t],
          N9[t], N10[t], N11[t], N12[t], N13[t], N14[t], N15[t], N16[t], N17[t]}, {t, 0, simTime}];
      {maxNuc, First[F / gamma /. posAns /. t → 480 // N]}, {maxNuc, 0, 200, 2}];
    ListPlot[{Speeds}, Joined → True, PlotRange → {{0, 200}, {0.0, 0.2}}]; (* remove the semi–colon to see the plot *)
```

Choose a Particular Nucleation Rate To Determine Exact Speed and Filament Populations

```
In[1366]:= maxNuc = 200; (* choose this value!! *)
bEqs = Table[{B1[t], B2[t], B3[t], B4[t], B5[t], B6[t],
    B7[t], B8[t], B9[t], B10[t], B11[t], B12[t], B13[t], B14[t], B15[t], B16[t], B17[t]}];
nEqs = Table[{N1[t], N2[t], N3[t], N4[t], N5[t], N6[t], N7[t], N8[t], N9[t], N10[t],
    N11[t], N12[t], N13[t], N14[t], N15[t], N16[t], N17[t]}];
posAns = NDSolve[{posEq, b1Eq, b2Eq, b3Eq, b4Eq, b5Eq, b6Eq, b7Eq, b8Eq, b9Eq, b10Eq, b11Eq, b12Eq,
    b13Eq, b14Eq, b15Eq, b16Eq, b17Eq, posEqInit, b1EqInit, b2EqInit, b3EqInit, b4EqInit, b5EqInit,
    b6EqInit, b7EqInit, b8EqInit, b9EqInit, b10EqInit, b11EqInit, b12EqInit, b13EqInit, b14EqInit, b15EqInit,
    b16EqInit, b17EqInit, n1Eq, n2Eq, n3Eq, n4Eq, n5Eq, n6Eq, n7Eq, n8Eq, n9Eq, n10Eq, n11Eq, n12Eq,
    n13Eq, n14Eq, n15Eq, n16Eq, n17Eq, n1EqInit, n2EqInit, n3EqInit, n4EqInit, n5EqInit, n6EqInit, n7EqInit,
    n8EqInit, n9EqInit, n10EqInit, n11EqInit, n12EqInit, n13EqInit, n14EqInit, n15EqInit, n16EqInit, n17EqInit},
   {x[t], B1[t], B2[t], B3[t], B4[t], B5[t], B6[t], B7[t], B8[t], B9[t], B10[t], B11[t], B12[t], B13[t],
    B14[t], B15[t], B16[t], B17[t], N1[t], N2[t], N3[t], N4[t], N5[t], N6[t], N7[t], N8[t],
    N9[t], N10[t], N11[t], N12[t], N13[t], N14[t], N15[t], N16[t], N17[t]}, {t, 0, simTime}];

(* Plot some observables.... remove the semi-colon at the end of plot statements to see the graphics *)
plotSize = 400;
Plot[Evaluate[ForceElements /. posAns], {t, 0, simTime}, PlotRange → {{0, simTime}, {0, 400}},
  AxesLabel → {"time (s)        ", "                    b-ends pushing the bacterium          "}, ImageSize → plotSize];
Plot[Evaluate[F / gamma /. posAns], {t, 0, simTime}, PlotRange → {{0, simTime}, {0, .3}},
  AxesLabel → {"time (s)        ", "                    speed (μm/s)                          "}, ImageSize → plotSize];
finalTipsCts = Table[First[bEqs[[i]] /. posAns /. t → 480 // N], {i, 1, elementNum}];
finalActinCts = Table[First[nEqs[[i]] /. posAns /. t → 480 // N], {i, 1, elementNum}];
ListPlot[finalTipsCts, Joined → True, AxesLabel →
   {"position on bug (bin)", "barbed-end count at steady-state in each element"}, ImageSize → plotSize];
ListPlot[finalActinCts, Joined → True, AxesLabel → {"position on bug (bin)",
    "  actin quantity at steady-state in each element  "}, ImageSize → plotSize];

ssSpeed = First[ F / gamma /. posAns /. t → 480 // N];
ssTips = First[ForceElements /. posAns /. t → 480 // N];
ssActin = First[DragElementsActin /. posAns /. t → 480 // N];

Print["RESULTS:"]
Print["Steady-state speed = ", ssSpeed, " μm/s"]
Print["Steady-state number of filament barbed-ends pushing the bacterium = ", ssTips]
Print["Steady-state actin measure = ", ssActin]
```

```
RESULTS:

Steady-state speed = 0.108055 μm/s

Steady-state number of filament barbed-ends pushing the bacterium = 141.949

Steady-state actin measure = 7340.62
```
