# Supplementary material for: An Experimental and Computational Study of the Effect of ActA Polarity on the Speed of Listeria monocytogenes Actin-based Motility
Source: PLoS Comput Biol. 2009 Jul 10;5(7):e1000434. doi: 10.1371/journal.pcbi.1000434 (PMC2699634; doi:10.1371/journal.pcbi.1000434)
Supplement: Table S1 — Concentrations used in the agent-based model (reproduced from Alberts and Odell 2004). These values are not for any specific cell type, but are typical biological concentrations and similar to those used for in vitro reconstitutions of bacterial motility. (0.02 MB DOC) [file pcbi.1000434.s009.doc]

| Entity | Concentration (µM) | Source |
| --- | --- | --- |
| Unpolymerized actin | 12 | Pollard et al. 2000 |
| ADF/Cofilin | 3 | Table 1 values for *Xenopus* extract |
| Profilin | 5 |  |
| Arp2/3 complex | 0.3 | Pollard et al. 2000 |
| Capping protein | 1 | Table 1 typical values |
| VASP | 0.5 | Loisel et al. 1999 |
| ActA | 10^5^/µm^2^ | J. Theriot, personal communication |
