# Supplementary material for: An Experimental and Computational Study of the Effect of ActA Polarity on the Speed of Listeria monocytogenes Actin-based Motility
Source: PLoS Comput Biol. 2009 Jul 10;5(7):e1000434. doi: 10.1371/journal.pcbi.1000434 (PMC2699634; doi:10.1371/journal.pcbi.1000434)
Supplement: Table S2 — Rates used in the agent-based model (reproduced from Alberts and Odell 2004). A hydrolysis rate is given for a vectorial ATP hydrolysis model; experimental evidence currently supports the random hydrolysis model but we have, for simplicity, implemented a vectorial scheme for this analysis. That is, we assume that there is a distinct border within each filament between the ATP actin, ADP-Pi actin, and ADP actin regions; only monomers adjacent to these borders can transition from ATP actin to ADP-Pi actin or from ADP-Pi actin to ADP actin. We can readily switch to a random hydrolysis model in future studies. The values in angle brackets, for the interactions between ActA, Arp2/3, and actin monomers, are calculated considering the diffusive flux onto the bacterium's surface (see Alberts and Odell, 2004:Dataset S2). These values are thus dependent upon ActA density, the concentrations of Arp2/3 and actin, and a heuristic adjustment of these rates to balance new filament nucleation and side-branching in order to achieve realistic tail morphologies. The on-rates in brackets listed here apply to the concentrations in Table S1. (0.03 MB DOC) [file pcbi.1000434.s010.doc]

| Event | k_+_ (µM^-1^s^-1^) | k- (s^-1^) | K_d_ (µM) | Source |
| --- | --- | --- | --- | --- |
| Polymerization |  |  |  |  |
| ATP-actin, barbed end | 11.6 | 1.4 | 0.12 | Pollard 1986 |
| ADP-actin, barbed end | 3.8 | 7.2 | 1.9 |  |
| ATP-actin, pointed end | 1.3 | 0.8 | 0.62 |  |
| ADP-actin, pointed end | 0.16 | 0.27 | 1.7 |  |
| Capping | 3.0 | 4.0x10^-4^ | 1.0x10^-4^ | Schafter et al. 1996 |
| Hydrolysis |  |  |  |  |
| ATP🡪ADP-Pi actin |  | 12.3 |  | Carlier et al. 1987 |
| ADP-Pi🡪ADP actin |  | 0.0026 |  | Melki et al. 1996 |
| ActA interactions |  |  |  |  |
| ActA-Arp2/3 | <0.07> | 0.6 | <8.2> | Marchand et al. 2001 |
| ActA-Arp2/3-actin monomer  ActA-actin monomer | <0.4> | 30.0 | <77> | k_+_ values are concentration and geometry dependent |
